# Liposomal Nanoconfinement Enables Type I Photodynamic Conversion for Synergistic Cancer Photothermal‐Immunotherapy

**DOI:** 10.1002/advs.202515013

**Published:** 2026-02-15

**Authors:** Minglu Zhang, Shanshan Liang, Meng Wang, Tingfeng Zhang, Jinke Liu, Yuyuan Peng, Hao Fang, Lingna Zheng, Xiao He, Meng Wang, Luling Wu, Tony D. James, Wei Zhao, Bing Wang, Weiyue Feng

**Affiliations:** ^1^ Qingdao Central Hospital NHC Key Laboratory of Cardiopulmonary Rehabilitation and Functional Recovery Industry‐Academia‐Research Collaborative and Innovation Center For Intelligent Rehabilitation Drug R&D of Shandong Province Qingdao Key Laboratory of Precision Drug Research for Chronic Disease Rehabilitation School of Health and Life Sciences University of Health and Rehabilitation Sciences Qingdao China; ^2^ CAS Key Laboratory For Biomedical Effects of Nanomaterials and Nanosafety Institute of High Energy Physics Chinese Academy of Sciences Beijing China; ^3^ State Key Laboratory of Medicinal Chemical Biology College of Pharmacy Key Laboratory of Molecular Drug Research and KLMDASR of Tianjin Nankai University Tianjin China; ^4^ University of Chinese Academy of Sciences Beijing China; ^5^ State Key Laboratory of Analytical Chemistry For Life Science School of Chemistry Nanjing University Nanjing China; ^6^ Department of Chemistry University of Bath Bath BA27AY UK

**Keywords:** electron transfer, nanoconfinement, photoimmunotherapy, photothermal therapy, type I photodynamic therapy

## Abstract

Type‐I photodynamic therapy (PDT) agents offer a promising approach for treating hypoxic tumors. However, previous reports mainly focused on thermodynamic modulation through molecular engineering to block the Type‐II energy transfer pathway. Herein, we present a facile strategy to realize the conversion of Type II to Type I PDT by integrating liposomal confinement and electron/hydrogen transfer pathway. A multifunctional nanoplatform, **RhM‐R837@Lip** was developed, which facilitates an efficient shift from Type II to Type I for hemicyanine‐based photosensitizer (PS) by suppressing singlet oxygen (^1^O_2_) generation while promoting superoxide anion (O_2_^•−^) and hydroxyl radical (•OH). Lipids serve as electron donors, facilitating electron transfer to form PS radical anions. Additionally, liposomal nanoconfinement acts as a photothermal nanoreactor, achieving a photothermal conversion efficiency as high as 56.1%. Co‐encapsulation of immunoadjuvant R837 stimulates systemic immune responses, synergistically enhancing tumor eradication. This radical‐switching behavior, driven by liposomal nanoconfinement and the donor–π–acceptor (D‐π‐A) structural configuration, modulates electron transfer pathways to favor Type‐I photoreactions. The **RhM‐R837@Lip** nanoplatform provides a versatile, integrated strategy to overcome hypoxic tumor microenvironments, improving PDT and photocatalytic performance, and effectively inhibits tumor metastasis.

## Graphical abstract

Liposomal nanoconfinement converts the hemicyanine photosensitizer RhM from Type‐II to Type‐I photodynamic therapy (PDT) while enhancing photothermal therapy (PTT). The RhM‐R837@Lip nanoassembly efficiently generates superoxide (O_2_•^−^) and hydroxyl radicals (•OH) to kill tumor cells and achieve tumor ablation. Moreover, liposomes co‐deliver the immunoadjuvant R837, inducing immunogenic cell death (ICD) and activating systemic antitumor immunity.

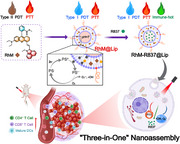

## 1 Introduction

Phototherapy has emerged as a promising cancer treatment modality due to its non‐invasive nature, minimal systemic toxicity, lack of resistance, and high spatiotemporal precision [1, 2, 3, 4, 5, 6]. Among phototherapeutic approaches, photodynamic therapy (PDT), utilizes photosensitizers (PSs) to generate reactive oxygen species (ROS) through light‐triggered photochemical reactions, inducing cancer cell death, but Type II PDT clinical therapeutic effect is greatly restricted by tumor hypoxia [7, 8, 9, 10]. In contrast, Type I PDT involves electron or hydrogen atom transfer processes, generating superoxide anion (O_2_^•−^), hydrogen peroxide (H_2_O_2_) and hydroxyl radicals (•OH), offering superior efficacy in hypoxic tumors. This oxygen‐independent mechanism has spurred interest in Type I PDT as an alternative to Type II PDT [11, 12, 13, 14, 15, 16, 17]. Concurrently, photothermal therapy (PTT) utilizes photothermal agents (PTAs) to convert light energy into localized heat, thereby inducing apoptosis or necrosis independently of oxygen availability [18, 19, 20, 21]. Thus, combining Type I PDT and PTT offers a synergistic approach for effective tumor ablation [22, 23]. To develop efficient Type I PSs, molecular design strategies such as heavy atom incorporation [24], π‐conjugation enhancement [25], polymerization [13], aggregation‐induced emission [26], and Förster energy transfer have been explored [27]. Similarly, PTA performance has been optimized by modulating intramolecular rotations and intermolecular interactions [28]. However, photoexcited molecules distribute energy among three main competing processes, namely fluorescence emission, intersystem crossing for PDT, and non‐radiative vibrational relaxation for PTT, in ways that are challenging to predict and or control [29]. This unpredictability, coupled with limited rational design strategies, hinders the systematic development of multifunctional phototherapeutic agents. Moreover, the photophysical behavior of PSs is highly sensitive to the microenvironment, which significantly influences ROS generation and photothermal conversion efficacy in vivo [30, 31, 32]. Despite this, little attention has been devoted to the microenvironment to optimize PS performance, particularly the transition from Type II to Type I mechanisms [33, 34, 35, 36]. Strategically modulating the PS microenvironment to achieve both Type I PDT and PTT holds significant potential for synergistic cancer therapy.

In parallel, tumor immunotherapy has gained prominence for inhibiting tumor progression, metastasis, and recurrence by stimulating the host immune responses. It offers high selectivity, reduced systemic toxicity, and long‐lasting memory [37, 38, 39]. Notably, phototherapy can induce immunogenic cell death (ICD), triggering the release of tumor‐associated antigens and damage‐associated molecular patterns such as calreticulin (CRT) exposure, high mobility group box protein 1 (HMGB1) release, and ATP secretion, which activate anti‐tumor immunity [40, 41, 42]. However, phototherapy alone often fails to elicit an sufficient immune response. Co‐delivery of immune adjuvants, such as Toll‐like receptor (TLR) agonists, can enhance dendritic cell (DC) maturation and antigen presentation, amplifying the immune response [43, 44]. For instance, imiquimod (R837), a TLR7/8‐specific agonist, effectively activate DCs to stimulate immunity [45, 46, 47, 48]. To harness the full potential of phototherapy and immunotherapy, we propose a “three‐in‐one” multifunctional platform integrating Type I PDT, PTT, and immune activation to achieve tumor eradication and systemic anti‐tumor immunity.

**SCHEME 1:**
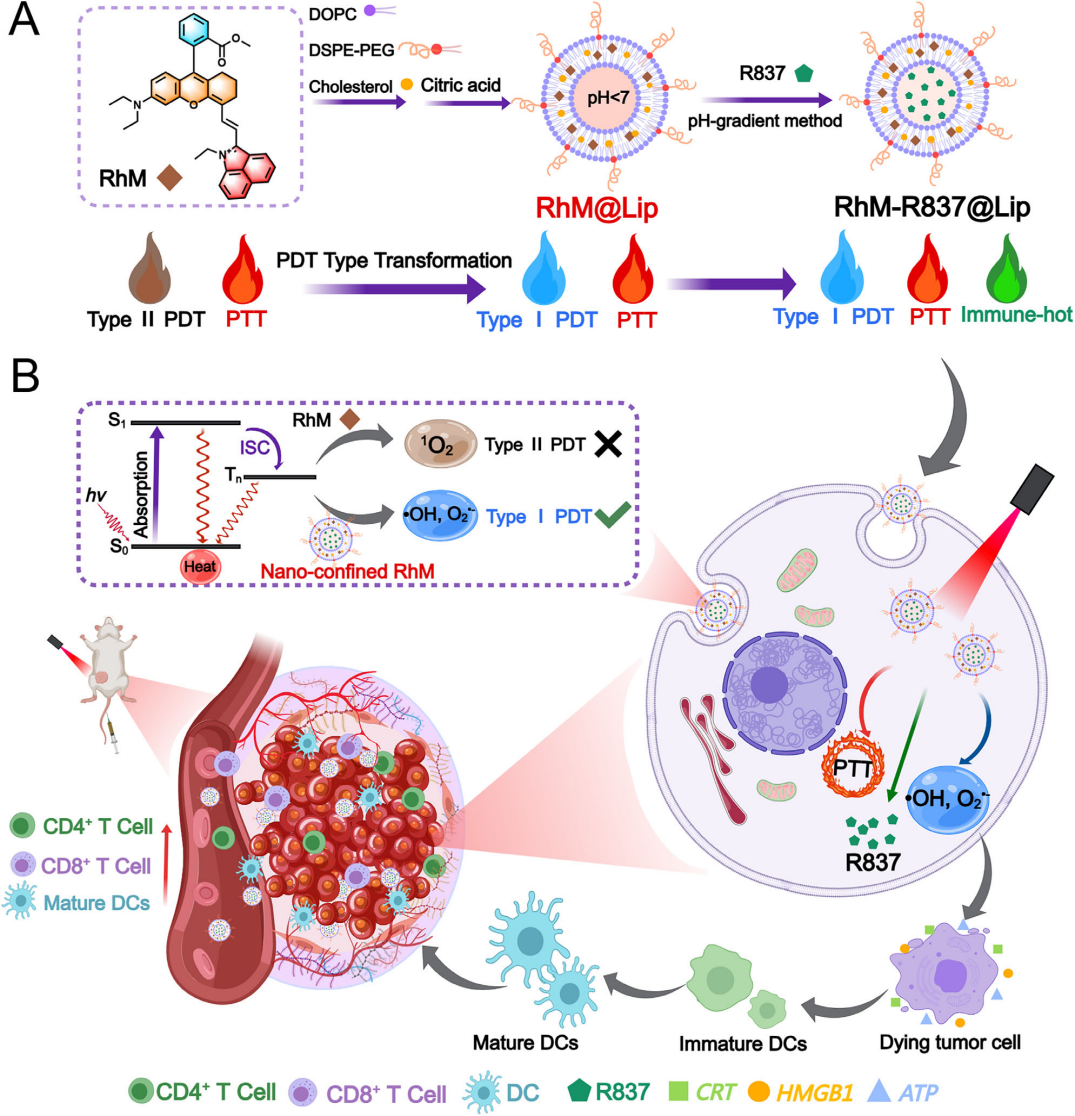
Design of **RhM‐R837@Lip** for synergistic tumor therapy. (A) Schematic illustration of the **RhM‐R837@Lip** assembly process and its transition from Type II to Type I photodynamic therapy (PDT). (B) Internalization of **RhM‐R837@Lip** into cancer cells via endocytosis, enabling Type I PDT and photothermal therapy (PTT) to induce tumor cell death while enhancing immune infiltration in tumor tissues.

In this study, we developed a novel liposome‐confined hemicyanine‐based nanoassembly, **RhM‐R837@Lip**, for synergistic photodynamic, photothermal, and immunotherapy (Scheme 1). A benz[c,d]indolium‐substituted hemicyanine derivative, **RhM**, based on our previous work [49], serves as a dual‐function photosentizier with intrinsic Type II PDT and PTT capabilities. Upon encapsulation into liposomes (**RhM@Lip**), **RhM** undergoes nano‐confinement, resulting in: i) strong near‐infrared absorption centered at 840 nm, compatible with 808 nm laser excitation; ii) a shift from Type II to Type I ROS generation; and iii) enhanced photothermal conversion efficacy. Additionally, the immune adjuvant R837 was incorporated using a pH‐gradient method, forming **RhM‐R837@Lip**, a multifunctional nanoassembly combining immunostimulation with dual‐modality phototherapy. Upon systemic administration, **RhM‐R837@Lip** preferentially accumulates at tumor sites via the enhanced permeability and retention effect. Subsequent laser irradiation initiates Type I PDT and PTT, causing extensive tumor cell damage and ICD, marked by the release of HMGB1, CRT, and ATP, thereby potentiating anti‐tumor immune responses. In summary, this work presents a rationally designed multifunctional nanoplatform that integrates phototherapy and immunotherapy to overcome the limitations of conventional PDT in hypoxic tumors, offering a promising strategy for durable and systemic anti‐tumor effects.

## 2 Results and Discussion

### 2.1 Preparation and Characterization of RhM‐R837@Lip

**FIGURE 1:**
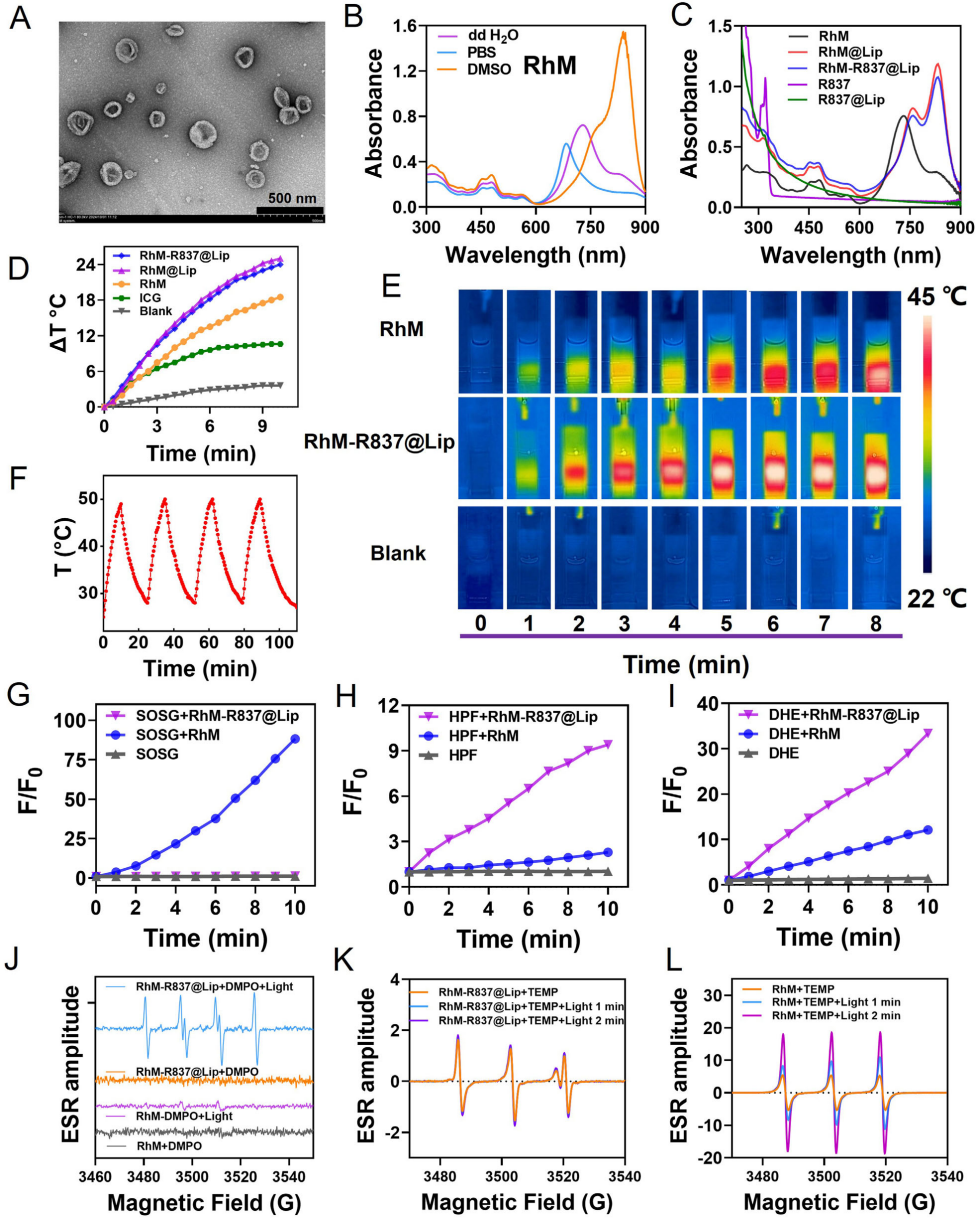
Characterization and photophysical properties of **RhM‐R837@Lip**. (A) Transmission electron microscopy image of **RhM‐R837@Lip**. Scale bar: 500 nm. (B) UV–vis absorbance spectra of **RhM** (20 µm) in different solvents. (C) UV–vis absorbance spectra of **RhM**, R837, **RhM@Lip**, **R837@Lip**, and **RhM‐R837@Lip** in PBS. (D) Temperature changes of free **RhM**, ICG, **RhM@Lip**, and **RhM‐R837@Lip** (**RhM** 20 µm, R837:**RhM** mass ratio = 2:1) under 808 nm NIR laser irradiation for 10 min (1 W/cm^2^). (E) Infrared thermal images of free **RhM** and **RhM‐R837@Lip** (**RhM** 20 µm, R837:**RhM** mass ratio = 2:1) under 808 nm laser irradiation (1 W/cm^2^) at different time points (0‐8 min). (F) Temperature variations of **RhM‐R837@Lip** (20 µm) over four on/off irradiation cycles. (G) Plots of relative fluorescence intensity (F/F_0_) at 525 nm of Singlet Oxygen Sensor Green (SOSG, 5 µm) to monitor ^1^O_2_ generation by **RhM** and **RhM‐R837@Lip** (**RhM** 10 µm, R837:**RhM** mass ratio = 2:1) under 808 nm irradiation (0.5 W/cm^2^), Ex: 504 nm. (H) Plots of relative fluorescence intensity (F/F_0_) at 515 nm of HPF (40 µm) to monitor •OH generation by **RhM** and **RhM‐R837@Lip** (**RhM** 10 µm, R837:**RhM** mass ratio = 2:1), Ex: 490 nm. (I) Plots of relative fluorescence intensity (F/F_0_) at 590 nm of DHE (40 µm) to monitor O_2_^•−^ generation by **RhM** and **RhM‐R837@Lip** (**RhM** 10 µm, R837:**RhM** mass ratio = 2:1), Ex: 490 nm. (J) ESR spectra of the mixture containing **RhM** or **RhM‐R837@Lip** and DMPO under light irradiation. (K) ESR spectra detecting ^1^O_2_ generated by **RhM‐R837@Lip** (0.5 mm) under light irradiation using TEMP as the spin‐trapping agent. (L) ESR spectra detecting ^1^O_2_ generated by **RhM** (0.5 mM) under light irradiation using TEMP as the spin‐trapping agent.

The synthesis of **RhM‐R837@Lip** is schematically depicted in Supporting Information. Briefly, a hemicyanine dye with donor‐π‐acceptor (D‐π‐A) structure, termed **RhM**, was synthesized (Scheme S1) and characterized (Figures S1–S8). Subsequently, **RhM**‐loaded liposomes (**RhM@Lip**) were prepared via thin‐film hydration, followed by remote loading of the TLR7/8 agonist imiquimod (R837) to form **RhM‐R837@Lip** (Scheme S2). Dynamic light scattering (DLS) and transmission electron microscopy revealed that **RhM‐R837@Lip** forms spherical vesicles with an average diameter of approximately 130 nm (Figure 1A; Figure S9). The encapsulation efficiency and drug loading of **RhM** were 91.2 ± 6.8% and 1.17 ± 0.35 wt.%, respectively, as determined by UV–vis spectroscopy. For R837, encapsulation efficiency and drug loading, measured via high‐performance liquid chromatography, were 43.8 ± 5.2%, and 2.23 ± 0.37 wt.%, respectively (Figure S10). Notably, free **RhM** tends to self‐aggregate in aqueous solution (∼120 nm, Figure S11) with a maximum absorption peak at 740 nm (Figure 1B). In contrast, the UV–vis spectra of **RhM@Lip** and **RhM‐R837@Lip** in phosphate‐buffered saline (PBS) exhibited a pronounced redshift of the maximum absorption peak to 840 nm (Figure 1C), consistent with the spectrum of free **RhM** in DMSO. This indicates that the liposomal formulation provides a lipophilic microenvironment, preventing **RhM** aggregation under physiological conditions and preserving its favorable near‐infrared (NIR) optical properties. Owing to liposomal encapsulation, the molar extinction coefficient of **RhM‐R837@Lip** reached 5.91×10^4^ M^−1^ cm^−1^, approximately twice that of free **RhM** (3.63 × 10^4^ M^−1^ cm^−1^, Figure S12), thereby enhancing its potential for deep‐tissue phototherapy.

### 2.2 In Vitro Photophysical Properties

To evaluate photothermal effects, temperature changes were monitored under 808 nm irradiation. **RhM@Lip** and **RhM‐R837@Lip** induced a temperature increase of ∼25°C in 10 min, significantly higher than that of free **RhM** (18°C), demonstrating enhanced photothermal conversion likely due to increased light absorption within the lipid bilayer (Figure 1D,E; Figure S13). Compared to the gold nanorods reference agent (87.1%), **RhM‐R837@Lip** exhibited a photothermal conversion efficiency of 56.1% (Figure S14). Additionally, owing to its strong photothermal capability, **RhM** was benchmarked against the cyanine photothermal agent indocyanine green (ICG). Under identical irradiation conditions, both **RhM** and **RhM‐R837@Lip** generated a higher temperature elevation than ICG (Figure 1D), demonstrating the superior photothermal performance and photostability of **RhM** compared to conventional cyanine dyes. Furthermore, we assessed the thermal stability of **RhM‐R837@Lip** by measuring DLS and UV–vis absorption spectra before and after laser irradiation. As shown in Figure S15, **RhM‐R837@Lip** displayed no noticeable changes in particle size or absorption peak position after irradiation and maintained stable photothermal performance over four on/off irradiation cycles (Figure 1F). These results indicate that the liposomes remain structurally intact and are not disrupted by the photothermal heating of the encapsulated photosensitizer.

To assess photodynamic activity, singlet oxygen sensor green (SOSG), hydroxyphenyl fluorescein (HPF), and dihydroethidium (DHE) were used to monitor ^1^O_2_, •OH, and O_2_^•−^ generation, respectively. Free **RhM** showed a strong SOSG fluorescence enhancement (88.2‐fold) at 525 nm, indicating predominant ^1^O_2_ generation. In contrast, **RhM@Lip** and **RhM‐R837@Lip** exhibited negligible SOSG fluorescence changes, suggesting reduced Type II PDT activity (Figure 1G; Figure S16). However, **RhM‐R837@Lip** induced stronger HPF and DHE fluorescence signals (9.4‐ and 33.4‐fold increases, respectively) compared to free **RhM** (2.1‐ and 12.1‐fold), indicating enhanced generation of •OH, and O_2_^•−^ (Figure 1H,I). These results indicate a mechanistic shift of photosensitizer **RhM** from Type II PDT to Type I PDT upon liposomal encapsulation. To evaluate and compare the ROS generation abilities of **RhM**, **RhM‐R837@Lip**, and NIR photosensitizer ICG, we employed 1,3‐diphenylisobenzofuran (DPBF) as a ROS indicator. The results showed that **RhM‐R837@Lip** generated markedly higher levels of ROS than ICG under identical conditions (Figure S17).

Electron spin resonance (ESR) spectroscopy was further performed to confirm that **RhM** transitions from Type‐II to Type‐I photodynamic pathways after liposomal encapsulation. 2,2,6,6‐Tetramethyl‐4‐piperidone hydrochloride (TEMP, for ^1^O_2_ detection) and 5,5‐dimethyl‐1‐pyrroline N‐oxide (DMPO, for O_2_^•−^ detection) were used as spin‐trapping agents. A clear ESR signal corresponding to the DMPO–O_2_^•−^ adduct was observed in the **RhM‐R837@Lip** group only under light irradiation, confirming the formation of Type‐I radicals (Figure 1J). As shown in Figure 1K,L, a three‐line ESR signal of TEMP–^1^O_2_ adduct appeared in the **RhM** group upon light irradiation, whereas no such signal was detected for **RhM‐R837@Lip**. These results directly demonstrate that **RhM‐R837@Lip** predominantly operates through a radical‐mediated Type‐I PDT pathway rather than a ^1^O_2_‐driven Type‐II mechanism, and that liposomal encapsulation effectively redirects **RhM** toward Type I photoreactivity.

Femtosecond transient absorption spectroscopy was then employed to elucidate the excited‐state dynamics of **RhM** and **RhM‐R837@Lip**. For **RhM‐R837@Lip**, a broad excited‐state absorption (ESA) band centered at ∼622 nm (τ = 29.21 ps) and a ground‐state bleaching (GSB) signal at ∼835 nm (τ = 34.24 ps) were observed, indicative of a predominantly short‐lived singlet‐state relaxation process under liposomal nanoconfinement. In contrast, free **RhM** exhibited additional features, including a distinct GSB at ∼675 nm and a long‐lived ESA band at ∼1090 nm (Figure S18). Upon encapsulation, both the 675 nm GSB band and the long‐lived 1090 nm ESA completely disappeared, demonstrating that nanoconfinement substantially restructures the excited‐state relaxation landscape of **RhM** (Figure S19). This change is likely associated with nanoconfinement‐induced alterations in molecular aggregation, thereby redefining deactivation pathways and influencing subsequent photochemical reactivity.

Liposomes serve as electron donors, with the flexibly conjugated **RhM** embedded in the phospholipid bilayer, enabling intimate contact with surrounding lipid molecules [50]. Under laser irradiation, the triplet excited state of **RhM** directly abstracts electrons from lipids, generating **RhM** radical anions and lipid radicals. These reactive intermediates subsequently react with molecular oxygen to efficiently produce Type I reactive oxygen species. Additionally, the liposomal bilayer creates a hydrophobic nanocage structure that ensures homogeneous dispersion of photosensitizers and enhances photodynamic performance [51, 52]. Similar enhancements were observed for other hydrophobic photosensitizer (Hypocrellin B) in aqueous solutions upon liposomal encapsulation (Figure S20). In summary, liposomes provide a lipophilic microenvironment for hydrophobic photosensitizers, significantly enhancing their photophysical properties and offering a versatile platform for optimized phototherapy applications.

### 2.3 In Vitro Cell Uptake and Subcellular Localization of RhM‐R837@Lip

**FIGURE 2:**
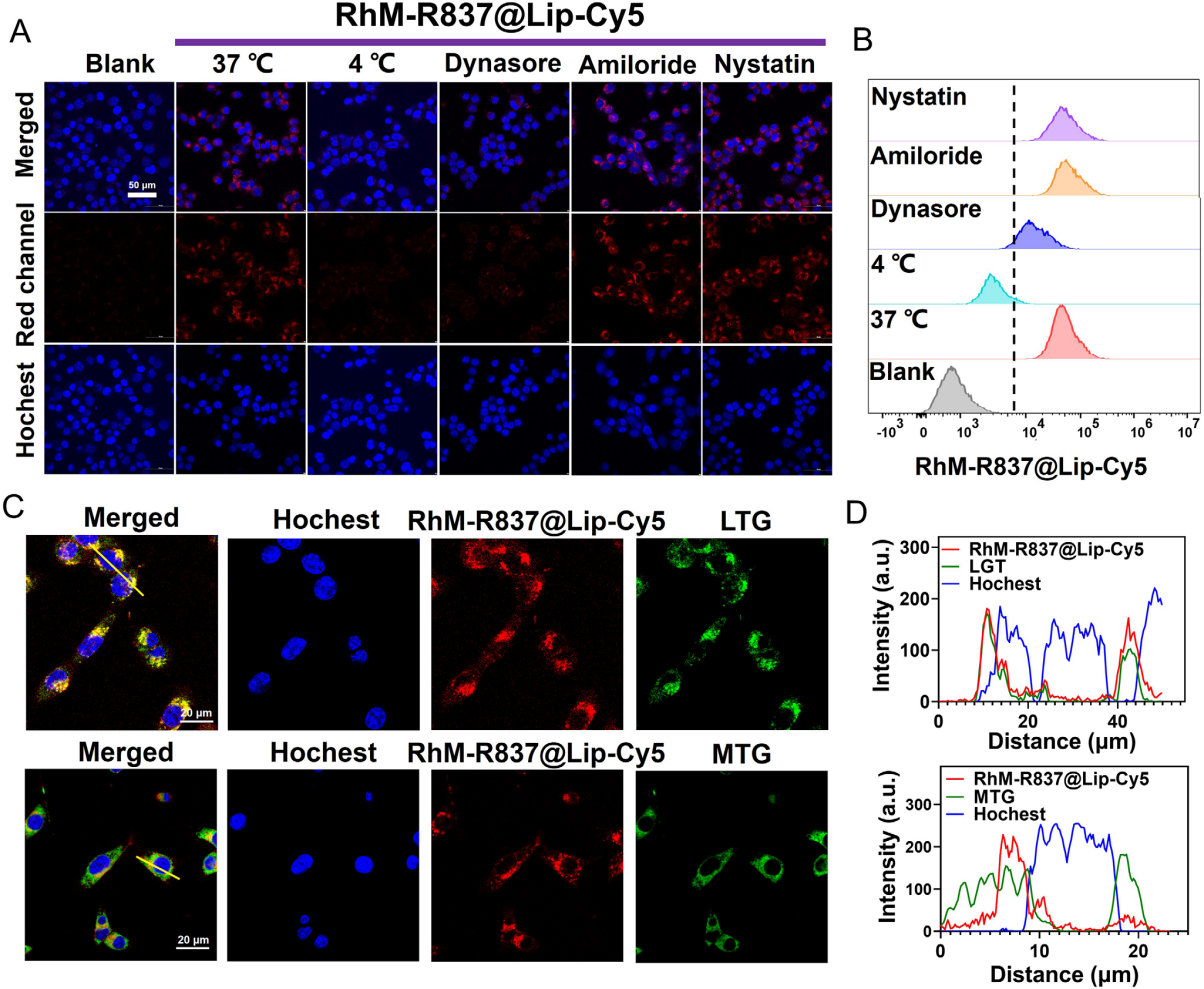
Cell uptake and subcellular localization of **RhM‐R837@Lip**. (A) Uptake behavior of **RhM‐R837@Lip‐Cy5** (**RhM** 0.5 µm, mass ratio of R837 to **RhM** at 2:1) was observed by Confocal laser scanning microscopy (CLSM). Scale bar: 50 µm. Blue channel: Ex = 405 nm, collected from 430‐475 nm. Red channel: Ex = 640 nm, collected from 663‐738 nm. (B) Flow cytometric analysis of uptake of 4T1 cells treated under low‐temperature conditions or with various endocytosis inhibitors. (C) Colocalization images of **RhM‐R837@Lip‐Cy5** (**RhM** 0.5 µm)‐treated cells with LysoTracker Green or MitoTracker Green. Scale bar: 20 µm. Blue channel: Ex = 405 nm, collected from 430‐475 nm. Green channel: Ex = 488 nm, collected from 500‐550 nm. Red channel: Ex = 640 nm, collected from 663‐738 nm. (D) Subcellular colocalization analysis of (C).

The intracellular uptake and subcellular localization of PSs are crucial for PDT efficiency, as ROS have a short half‐life and limited diffusion distance, exerting cytotoxic effects primarily near their generation sites. Hydrophobic PSs often exhibit limited aqueous solubility and cellular internalization, typically entering into cells via passive diffusion or remaining on the cell surface. In contrast, liposomes serve as advanced nanocarriers that encapsulate hydrophobic PSs, enhancing their physicochemical stability, bioavailability, and intracellular delivery efficiency. Since **RhM‐R837@Lip** lacks intrinsic fluorescence, fluorescently labeled analogs were prepared by substituting DSPE‐PEG 2000 in the liposomal formulation with DSPE‐PEG_2k_‐Cy5 or DSPE‐PEG_2k_‐Cy7, yielding **RhM‐R837@Lip‐Cy5** and **RhM‐R837@Lip‐Cy7** respectively (Scheme S3). These labeled formulations were used in cellular uptake, subcellular colocalization, and time‐dependent accumulation studies to infer the intracellular behavior of **RhM‐R837@Lip**. Although not chemically identical, these fluorescent analogs are widely accepted as reliable proxies for evaluating the intracellular fate of liposome‐based drug delivery systems. To investigate the cellular uptake pathway of **RhM‐R837@Lip**, 4T1 cells were incubated with **RhM‐R837@Lip‐Cy5** and analyzed using confocal laser scanning microscopy and flow cytometry. Cellular internalization was further evaluated under low‐temperature conditions and in the presence of various endocytosis inhibitors. Specifically, 4T1 cells were pretreated for 1 h with dynasore (a clathrin‐mediated endocytosis inhibitor), amiloride hydrochloride (a macropinocytosis inhibitor), and nystatin (a caveolin‐mediated endocytosis inhibitor). As shown in Figure 2A,B the uptake of **RhM‐R837@Lip‐Cy5** was significantly reduced at 4°C and in the presence of dynasore, whereas only minimal inhibition was observed with amiloride and nystatin. These results indicate that **RhM‐R837@Lip** primarily enters 4T1 cells through an energy‐dependent clathrin‐mediated endocytosis pathway, which is consistent with previously reported mechanisms for liposome internalization [53].

Following endocytosis, liposomes are typically trafficked through endosomes and accumulate in lysosomes [54]. To assess the subcelluar localization of **RhM‐R837@Lip**, co‐staining experiments were conducted using MitoTracker Green (MTG) or LysoTracker Green (LTG). The fluorescence signal of **RhM‐R837@Lip‐Cy5** showed strong colocalization with LTG (Figure 2C,D), indicating predominant lysosomal accumulation. Upon laser exposure, **RhM‐R837@Lip** may induce lysosomal damage. Concurrently, the increase in local temperature and the generation of high levels of O_2_^•−^ and •OH may disrupt the liposome, facilitating the release of R837 encapsulated in the aqueous compartment. To determine the optimal incubation time for subsequent therapeutic studies, the time‐dependent internalization of **RhM‐R837@Lip‐Cy7** was monitored. The fluorescence intensity in 4T1 cells increased progressively and reached a maximum at 12 h (Figure S21), suggesting efficient accumulation and stability of the liposomal formulation within lysosomes. Based on these findings, a 12‐h incubation time was selected for all subsequent cellular experiments to achieve optimal Type I PDT performance.

### 2.4 In Vitro Anti‐Tumor Activity of RhM‐R837@Lip

**FIGURE 3:**
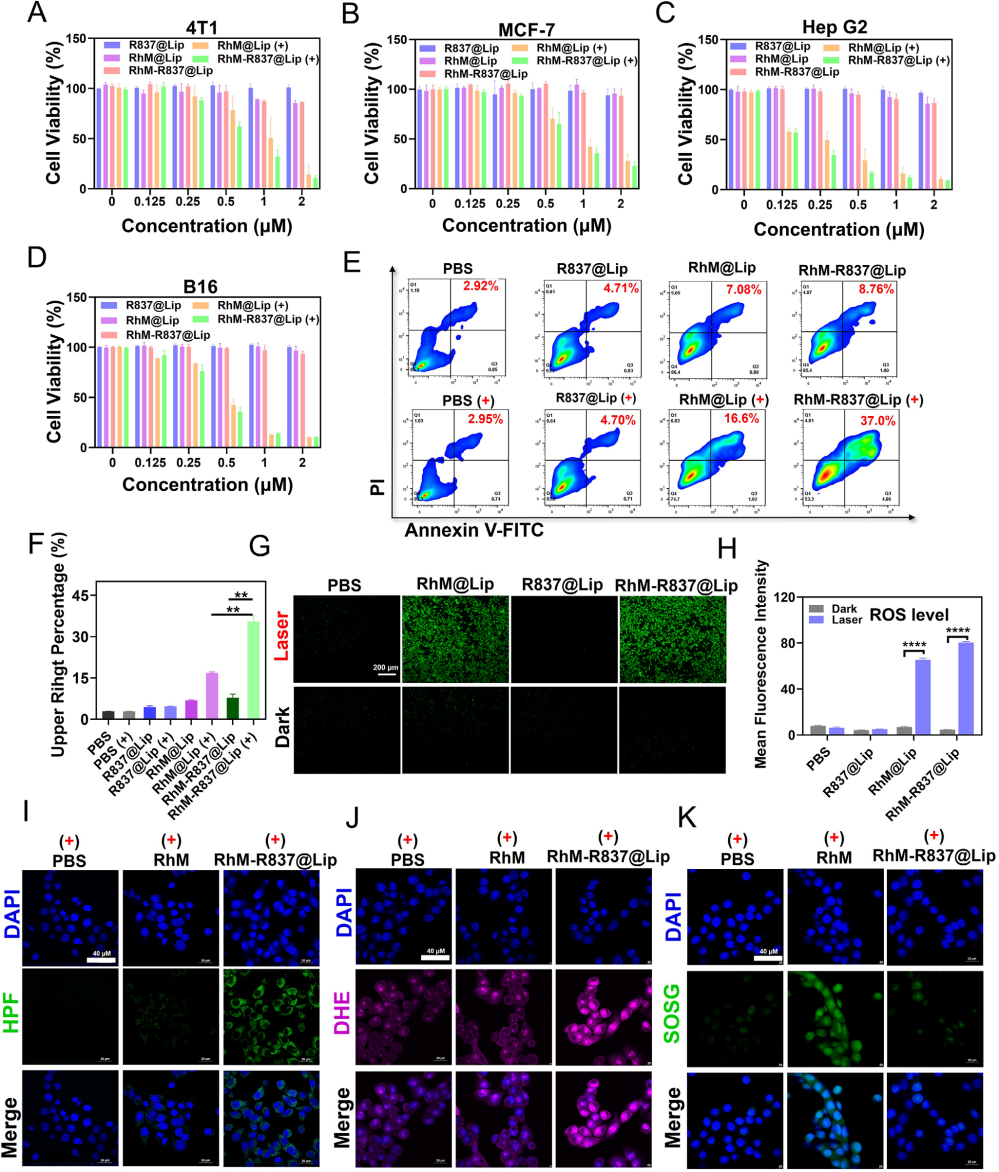
Photocytotoxic effects of **RhM‐R837@Lip**. (A‐D) Cell viability of 4T1, MCF‐7, Hep G2, and B16 cells treated with varying concentrations of **R837@Lip**, **RhM@Lip**, and **RhM‐R837@Lip** (**RhM** 0‐2 µm, mass ratio of R837 to **RhM** at 2:1) with or without NIR laser irradiation (0.5 W/cm^2^, 5 min). (+) indicates laser irradiation. (E) Flow cytometric analysis to detect apoptotic cells incubated with **R837@Lip**, **RhM@Lip**, and **RhM‐R837@Lip** (**RhM** 0.5 µm, mass ratio of R837 to **RhM** at 2:1) under NIR laser irradiation (0.5 W/cm^2^, 5 min). (+) indicates laser irradiation. (F) Statistical analysis of the upper right quadrant of (E). (G) CLSM images of 4T1 cells stained with DCFH‐DA and treated with **R837@Lip**, **RhM@Lip**, or **RhM‐R837@Lip** (**RhM** 0.5 µm, mass ratio of R837 to **RhM** at 2:1) under NIR laser irradiation (0.5 W/cm^2^, 5 min). Scale bar: 200 µm. Ex = 488 nm, collected from 500‐550 nm. (H) Mean fluorescence intensity of DCFH‐DA in (G). (I) Confocal fluorescence images of HPF stained 4T1 cells treated with PBS, **RhM**, or **RhM‐R837@Lip** followed by NIR laser irradiation (0.5 W/cm^2^, 5 min). Scale bar: 40 µm. Blue channel: DAPI, Ex = 405 nm, collected from 430‐475 nm. Green channel: HPF, Ex = 488 nm, collected from 500‐550 nm. (J) Confocal fluorescence images of DHE stained 4T1 cells treated with PBS, **RhM**, or **RhM‐R837@Lip** followed by NIR laser irradiation (0.5 W/cm^2^, 5 min). Scale bar: 40 µm. Blue channel: DAPI, Ex = 405 nm, collected from 430‐475 nm. Magenta channel: DHE, Ex = 561 nm, collected from 570‐616 nm. (K) Confocal fluorescence images of SOSG stained 4T1 cells treated with PBS, **RhM**, or **RhM‐R837@Lip** followed by NIR laser irradiation (0.5 W/cm^2^, 5 min). Scale bar: 40 µm. Blue channel: DAPI, Ex = 405 nm, collected from 430‐475 nm. Green channel: SOSG, Ex = 488 nm, collected from 500‐550 nm.

The in vitro anti‐tumor activity of **R837@Lip**, **RhM@Lip**, and **RhM‐R837@Lip** was evaluated using a Cell Counting Kit‐8 (CCK‐8) assay across a range of concentrations in 4T1, MCF‐7, Hep G2, and B16 cell lines. In addition, the cytotoxicity of **RhM@Lip** and **RhM‐R837@Lip** was assessed under 808 nm laser irradiation. All experiments were conducted based on the molar concentration of **RhM**, with a fixed mass ratio of R837 to **RhM** at 2:1 in the liposomal formulations. As shown in Figure 3A–D, **RhM@Lip** and **RhM‐R837@Lip** exhibited pronounced, concentration‐dependent cytotoxicity upon 808 nm laser exposure. In contrast, **R837@Lip**, **RhM@Lip**, and **RhM‐R837@Lip** showed negligible cytotoxicity in the absence of laser irradiation, indicating that **RhM** is essential for eliciting therapeutic effects upon light activation. Under the same conditions, **RhM‐R837@Lip** exhibited a markedly stronger cell‐killing effect than ICG and chlorin e6 (Figure S22). The biocompatibility of these formulations was further confirmed in normal cell lines including Chinese hamster lung (CHL) cells and human umbilical vein endothelial cells (HUVECs), where **RhM‐R837@Lip** exhibited no significant toxicity without laser exposure (Figure S23). Flow cytometric analysis revealed that **RhM‐R837@Lip**, upon laser 808 nm activation, induced approximately 37.0% late apoptosis or secondary necrosis (Annexin V^+^/PI^+^) in 4T1 cells, representing the most substantial cytotoxic response among all the tested groups. (Figure 3E,F).

To investigate intracellular ROS generation, 2’,7’‐dichlorofluorescin diacetate (DCFH‐DA) was used as a fluorescent probe. Following incubation with **RhM@Lip** or **RhM‐R837@Lip** and 808 nm laser irradiation, 4T1 cells exhibited a marked increase in green fluorescence intensity compared to other treatment groups (Figure 3G,H), indicating effective ROS production by light‐activated **RhM**, which contributes to cellular oxidative damage. In addition, to further elucidate the type of intracellular ROS generated, HPF, DHE, and SOSG were employed as fluorescent probes to detect •OH, O_2_^•−^, and ^1^O_2_, respectively. As shown in Figure 3I–K, distinct green fluorescence signals and magenta fluorescence signals could be observed in **RhM‐R837@Lip**‐treated cells after incubation with the probes followed by 808 nm irradiation, whereas weak fluorescence signals were observed in the **RhM** group and control group, indicating that **RhM‐R837@Lip** can generate •OH and O_2_^•−^ in cells under laser irradiation to kill cells through a Type‐I photodynamic mechanism. In contrast, when SOSG was used as a singlet oxygen indicator, only **RhM**‐treated cells exhibited strong green fluorescence, revealing that free **RhM** primarily follows a Type‐II photodynamic pathway in cells. These cellular results demonstrate that liposomal encapsulation redirects **RhM** from a Type‐II to a Type‐I photodynamic mechanism, ultimately enabling more effective tumor cell killing.

A live/dead cell co‐staining assay using Calcein acetoxymethyl ester (Calcein‐AM) and propidum iodide (PI) was subsequently performed. In the absence of irradiation, nearly all cells exhibited strong Calcein‐AM fluorescence, indicative of high viability. However, under 808 nm irradiation, intense red PI fluorescence was observed in the **RhM@Lip** and **RhM‐R837@Lip** groups (Figure S24), confirming the potent phototoxic effects of these formulations. Given that **RhM‐R837@Lip** predominantly operates through Type I PDT, which is less dependent on oxygen, this system is well‐suited for targeting hypoxic tumor microenvironments. Mitochondrial membrane potential (MMP) reduction is a hallmark of early‐stage apoptosis and often precedes irreversible cell death. To assess MMP disruption, 5, 5', 6, 6' ‐tetrachloro‐1, 1', 3, 3' ‐tetraethyl‐imidacarbocyanine iodide (JC‐1) staining assay was employed. Following 808 nm laser irradiation, both **RhM@Lip** and **RhM‐R837@Lip** treatments resulted in a marked increase in green fluorescence emission accompanied by a significant decrease in red fluorescence emission (Figure S25), indicating MMP decrease. This reduced MMP is characteristic of the initial phase of apoptosis and suggests mitochondrial photodamage was induced during the PDT and PTT processes. In summary, **RhM‐R837@Lip** demonstrated potent tumoricidal activity in vitro upon laser irradiation, with its primary cytotoxic effects attributed to the synergistic photodynamic and photothermal actions of the **RhM** component.

### 2.5 In Vitro Immune Response of RhM‐R837@Lip

**FIGURE 4:**
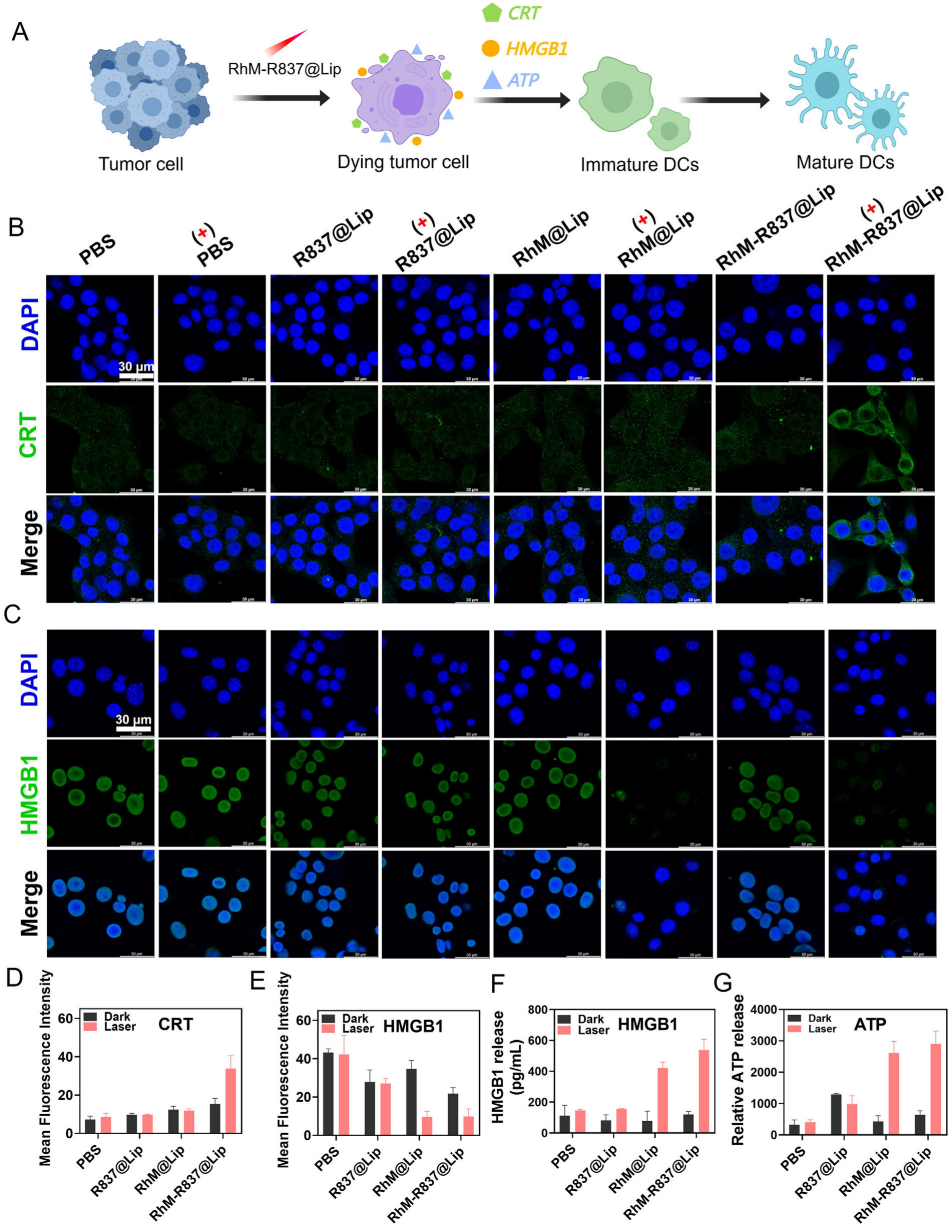
RhM**‐R837@Lip**‐mediated immunogenic cell death upon laser irradiation. (A) Schematic diagram illustrating **RhM‐R837@Lip** inducing the release of ICD‐associated damage‐associated molecular patterns and promoting DC maturation in vitro. (B) CLSM imaging of intracellular CRT (Green) in the different groups. “( + )” denotes samples exposed to laser irradiation. Scale bar: 30 µm. Blue channel: Ex = 405 nm, collected from 430–475 nm. Green channel: Ex = 488 nm, collected from 500–550 nm. (C) CLSM imaging of HMGB1 level (Green) in 4T1 cells after different treatments. Scale bar: 30 µm. Blue channel: Ex = 405 nm, collected from 430‐475 nm. Green channel: Ex = 488 nm, collected from 500–550 nm. (D) Mean fluorescence intensity analysis of CRT in (B). (E) Mean fluorescence intensity analysis of HMGB1 in (C). (F) HMGB1 levels in 4T1 cell culture supernatants after various treatments. (G) ATP levels in 4T1 cells supernatant after various treatments. Experimental conditions: **RhM** 0.5 µm, R837: **RhM** = 2:1 (mass ratio), 0.5 W/cm^2^ 808 nm laser irradiation for 8 min.

As shown in Figure 4A, phototherapy can induce the ICD, which involves the regulated release of damage‐associated molecular patterns (DAMPs) [55]. These DAMPs, including high mobility group box 1 (HMGB1), calreticulin (CRT), and adenosine triphosphate (ATP), facilitate the recruitment and activation of antigen‐presenting cells particularly DCs, ultimately triggering adaptive immunity [56]. To evaluate the potential of **RhM‐R837@Lip** as an ICD inducer, we assessed its effects on 4T1 cells. Figure 4B,D present the immunofluorescence results for CRT expression under various treatment conditions. The **RhM‐R837@Lip** group with 808 nm laser irradiation group exhibited the most pronounced green fluorescence, indicating its efficacy in inducing CRT expression in 4T1 cells. Subsequently, the release of HMGB1 in 4T1 cells was examined. Under dark conditions, HMGB1 (green fluorescence signal) in cells treated with **R837@Lip**, **RhM@Lip**, or **RhM‐R837@Lip** predominantly colocalized with the nucleus. In contrast, following **RhM‐R837@Lip** treatment with laser irradiation, HMGB1 was primarily translocated from the nucleus to the cytoplasm (Figure 4C,E). Furthermore, elevated levels of HMGB1 protein were detected in the cell culture supernatant by enzyme‐linked immunosorbent assay (ELISA) (Figure 4F). These findings suggest that **RhM‐R837@Lip**, under laser irradiation, significantly induces the extracellular release of HMGB1 in 4T1 cells. ATP levels in the supernatant of 4T1 cells after various treatments were quantified using a bioluminescence assay kit. As shown in Figure 4G, ATP secretion in the **RhM‐R837@Lip** group with laser irradiation was significantly higher compared to the other groups. Collectively, these results indicate that **RhM‐R837@Lip**, upon 808 nm laser irradiation, triggers ICD in tumor cells, which may activate an anti‐tumor immune response.

### 2.6 In Vivo Anti‐Tumor Effect of RhM‐R837@Lip

**FIGURE 5:**
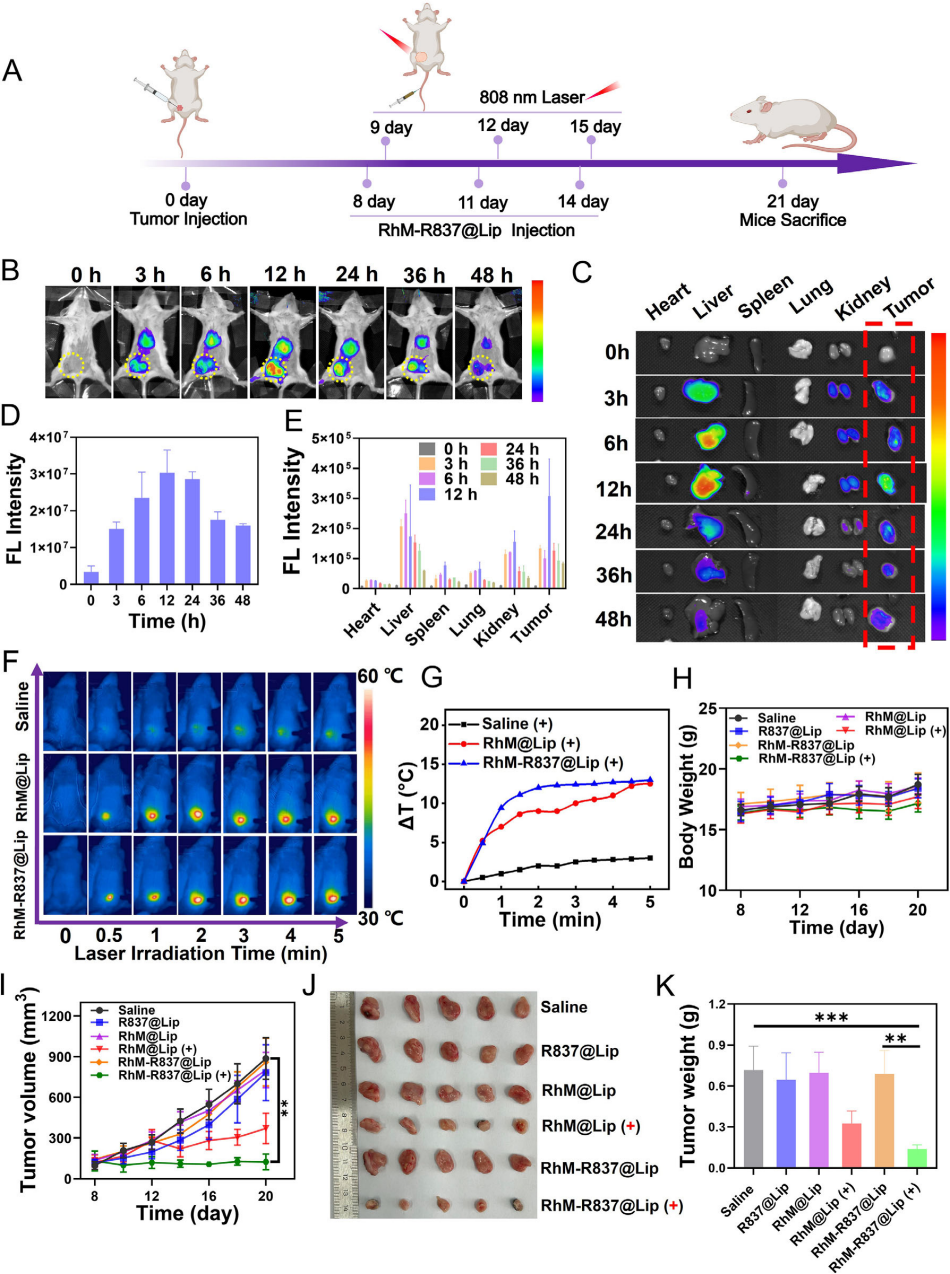
In vivo anti‐tumor activity of **RhM‐R837@Lip** in a 4T1 orthotopic tumor model. (A) Schematic illustration of the treatment regimen. (B) In vivo fluorescence images of mice intravenously injected with **RhM‐R837@Lip‐Cy7** (0.5 mg/kg **RhM**, 1 mg/kg R837) at the indicated time points. Ex:740 nm, Em = 760‐790 nm. (C) Ex vivo fluorescence images of excised organs and tumors collected at different time points. Ex:740 nm, Em = 760‐790 nm. (D) Quantification of fluorescence intensity in the tumor region of interest (yellow circle) over time. (E) Fluorescence intensity of organs and tumors at each time point. (F) Infrared thermal images of 4T1 tumor‐bearing mice subjected to 808 nm laser irradiation (0.5 W/cm^2^) after injection of **RhM‐R837@Lip** or **RhM@Lip** (**RhM** 0.5 mg/kg, R837 1 mg/kg). (G) Tumor‑site temperature elevation under the different treatments, “(+)” denotes laser irradiation (808 nm, 0.5 W/cm^2^). (H) Body‐weight curves of mice during treatment. (I) Tumor‐volume curves of mice for the various treatment groups. (J) Representative photographs of tumors harvested at the end of treatment. (K) Tumor weights at the study endpoint. Data are expressed as means ± SD (n = 5). Statistical significance was determined by Student's *t* test: *p < 0.05, **p < 0.01, and ***p < 0.001.

To evaluate the therapeutic effect of **RhM‐R837@Lip** in vivo, an orthotopic 4T1 tumor model was established in BALB/c mice (Figure 5A). We conducted a comprehensive assay about the organ distribution and tumor accumulation of **RhM‐R837@Lip** after intravenous injection at various time points via fluorescence labeled **RhM‐R837@Lip‐Cy7**. As shown in Figure 5B,D, the fluorescence intensity at the tumor site increased over time, reaching a maximum at 12 h post‐injection. Mice injected with **RhM‐R837@Lip** were euthanized at different time points, and organs and tumors were collected for in vivo imaging system analysis to examine the biodistribution. The fluorescence intensity was highest in the liver, the primary metabolic organ, followed by the tumor site, where the fluorescence intensity was also significantly elevated (Figure 5C,E). Confocal imaging of tumor sections showed that **RhM‐R837@Lip‐Cy7** was widely distributed within the tumor tissue (Figure S26). However, because tumor sections do not provide clear cell‐membrane delineation, this experiment cannot unambiguously distinguish intracellular from extracellular signals. Consistent with this, Desorption Electrospray Ionization–Mass Spectrometry Imaging (DESI‐MSI) further revealed a broad distribution of the **RhM** photosensitizer throughout the tumor microenvironment (Figure S27). This tumor accumulation may be attributed to the enhanced permeability and retention effect, which facilitates the internalization of **RhM‐R837@Lip** in the tumor region. These in vivo fluorescence images clarified the biodistribution, tumor‐targeting behavior of **RhM‐R837@Lip**, and indicated that 12 h post‐injection is the optimal window for laser irradiation.

The photothermal effects of **RhM‐R837@Lip** were then further assessed. For the groups receiving phototherapy, mice were intravenously injected with **RhM‐R837@Lip** (0.5 mg/kg **RhM**, 1 mg/kg R837) via the tail vein, followed by NIR laser irradiation (0.5 W/cm^2^) density 12 h post‐injection. As shown in Figure 5F,G, the tumor temperature in mice injected with **RhM‐R837@Lip** rose by approximately 13.5°C within 5 min, whereas the saline group showed an increase of only about 3°C. Since temperatures above 42°C can induce apoptosis or necrosis of tumor cells, these results indicate that **RhM‐R837@Lip** can effectively ablate tumors at the tested dose and laser intensity.

Body weight (Figure 5H) and tumor volume (Figure 5I) were monitored over a 14‐day period. Tumors in the saline group grew rapidly, while the **RhM‐R837@Lip** + laser group exhibited the most significant inhibition, with scarring observed at tumor sites (Figure S28). After 14 days, all mice were euthanized, and tumor tissues were harvested and weighed (Figure 5J,K). These results clearly demonstrate the potent tumor ablation efficacy of **RhM‐R837@Lip** under laser irradiation. Hematoxylin and eosin (H&E) staining revealed that tumor tissues from **RhM‐R837@Lip** + laser and **RhM@Lip** + laser groups displayed nuclear shrinkage, deformation, and loose density, indicating severe tissue damage. In contrast, tumors from the other groups remained densely packed (Figure S29). Ki‐67 immunohistochemical staining showed a significant decrease in cell proliferation in the **RhM@Lip** + laser and **RhM‐R837@Lip** + laser groups (Figure S30).

To investigate whether phototherapy could trigger anti‐tumor immune responses in vivo, we evaluated CRT exposure and HMGB1 protein in tumor tissues. As shown in Figure S31, CRT fluorescence intensity was markedly increased in the **RhM‐R837@Lip** + laser group compared to other groups. HMGB1 was localized in the nucleus in the saline group, whereas in the **RhM‐R837@Lip**+laser group, HMGB1 fluorescence no longer colocalized with Hoechst staining. These results confirm that **RhM‐R837@Lip** + laser treatment effectively induced ICD in vivo. Furthermore, immunofluorescence staining of CD3^+^/CD4^+^ and CD3^+^/CD8^+^ T cells (Figure S32) revealed the most significant T cell activation in the **RhM‐R837@Lip** + laser group among all treatments. These findings indicate that **RhM‐R837@Lip**‐mediated phototherapy not only induces ICD but also robustly activates an anti‐tumor immune responses.

### 2.7 In Vivo Systemic Anti‐Tumor Immune Response

Generally, photoimmunotherapy demonstrates superiority in treating both in situ tumors, metastasis, and cancer recurrence. To elicit a sufficient immune response against tumors, it is often necessary not only to reinvigorate the suppressed immune system through PDT/PTT‐induced immunogenic cell death but also to apply multiple stimuli to enhance tumor immunogenicity [1, 38, 57]. Accordingly, we combined the Toll‐like receptor agonist R837 with PDT/PTT, aiming to activate robust immune responses in vivo.

**FIGURE 6:**
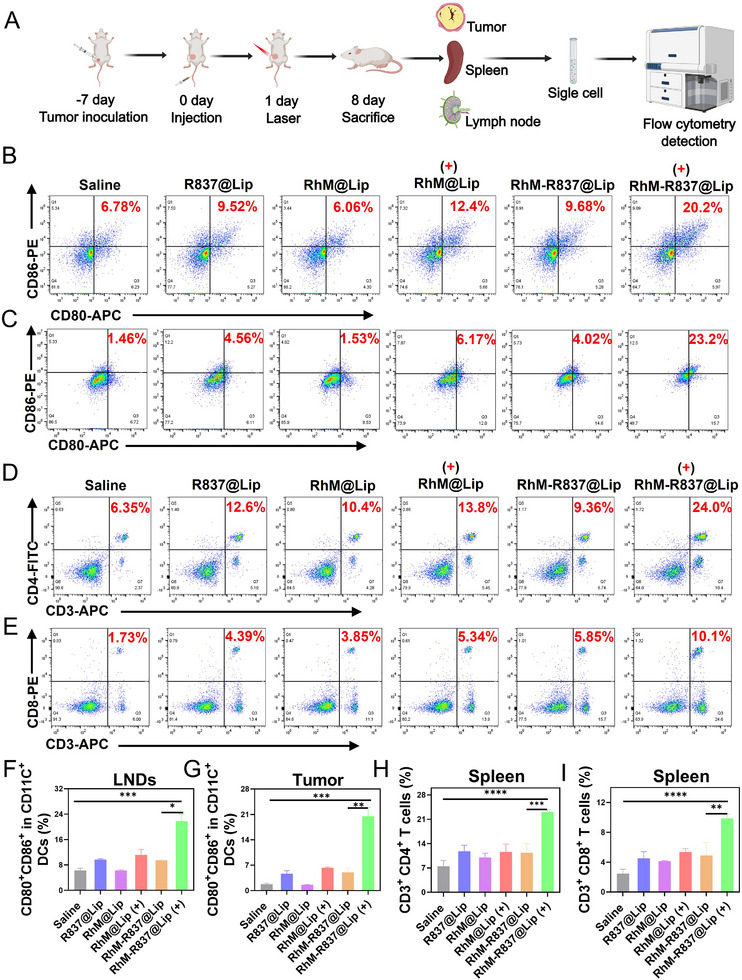
In vivo systemic anti‐tumor immune response triggered by **RhM‐R837@Lip**. (A) Schematic of anti‐tumor immune effects. **RhM** dose: 0.5 mg/kg; R837 dose: 1 mg/kg; laser: 808 nm, 0.5 W/cm^2^. (B) Mature DC (CD80^+^/CD86^+^) detection in inguinal lymph nodes of each group via flow cytometry. (C) Mature DC (CD80^+^/CD86^+^) detection in tumors of each group via flow cytometry. (D) Flow cytometric analysis of CD4^+^ T cells in the spleen. (E) Flow cytometric analysis of CD8^+^ T cells in the spleen. (F) Percentage of CD80^+^/CD86^+^ populations in inguinal lymph nodes. (G) Percentage of CD80^+^/CD86^+^ populations in tumors. (H) Population percentage of CD4^+^ T cells in the spleen. (I) Percentage of CD8^+^ T cells in spleen. Data are expressed as means ± SD (n = 3). Statistical significances were determined using Student's *t* test, *p < 0.05, **p < 0.01, ***p < 0.001 and ****p < 0.0001.

Encouraged by the aforementioned in vivo anti‐tumor experiments, we harvested tumor tissues and inguinal lymph nodes of mice to prepare single‐cell suspensions for flow cytometric analysis. As illustrated in Figure 6B,F, the proportion of mature DCs in the lymph nodes of the **RhM‐R837@Lip** + laser group was 2.9‐fold higher than that in the control group and significantly exceeded that in the **RhM@Lip** + laser and **R837@Lip** groups. In the tumor, the **RhM‐R837@Lip** + laser group also stimulated DC maturation more effectively. (Figure 6C,G). This observation suggests that the combination of **RhM** and the Toll‐like receptor agonist R837 plays a crucial role in inducing a robust anti‐tumor immune response.

Given that mature DCs can induce the generation of cytotoxic T lymphocytes and helper T lymphocytes, we subsequently investigated immune cell infiltration at the tumor site and systemic immune activation in the spleen. Tumor and spleen tissues were collected to quantify the levels of CD3^+^/CD4^+^ T cells and CD3^+^/CD8^+^ T cells. In the spleen, the **RhM‐R837@Lip** + laser group induced the expression of CD3^+^/CD4^+^ T cells at 3.8 times that of the control group (Figure 6D,H), and the expression of CD3^+^/CD8^+^ T cells at 5.8 times that of the control group (Figure 6E,I), both of which were higher than those in the **R837@Lip** group and the **RhM@Lip** + laser group. As illustrated in Figure S33, analysis of CD3^+^/CD4^+^ T cells and CD3^+^/CD8^+^ T cells in tumor suspensions from different treatment groups also revealed that the **RhM‐R837@Lip** + laser group exhibited the highest expression levels. Meanwhile, immunofluorescence analysis revealed that **RhM‐R837@Lip** under light irradiation markedly reduced the number of intratumoral CD4^+^CD25^+^Foxp3^+^ Treg cells (Figure S34). Consistently, cytokine analysis showed a pronounced increase in tumor necrosis factor‐α (TNF‐α) in the **RhM‐R837@Lip** (+) group, while interferon‐γ (IFN‐γ) levels were substantially upregulated across all irradiated groups (Figure S35). These changes in the tumor immune microenvironment indicate that **RhM‐R837@Lip** promotes a pro‐inflammatory and anti‐tumor immune response. In contrast, PD‐L1 expression exhibited minimal differences among the treatment groups (Figure S36), suggesting that the immunomodulatory activity of the combination therapy is primarily mediated through T‐cell activation and cytokine remodeling rather than through modulation of the PD‐1/PD‐L1 axis.

These results demonstrate that **RhM‐R837@Lip**‐mediated phototherapy exhibits superior immune activation efficacy compared to monotherapy with either **RhM@Lip** or **R837@Lip** in murine tumor models. The infiltration of these immune cells will contribute to the transformation of “cold” tumors to “hot” tumors, resulting in a systemic anti‐tumor effect.

### 2.8 Suppression of Distal Tumor

**FIGURE 7:**
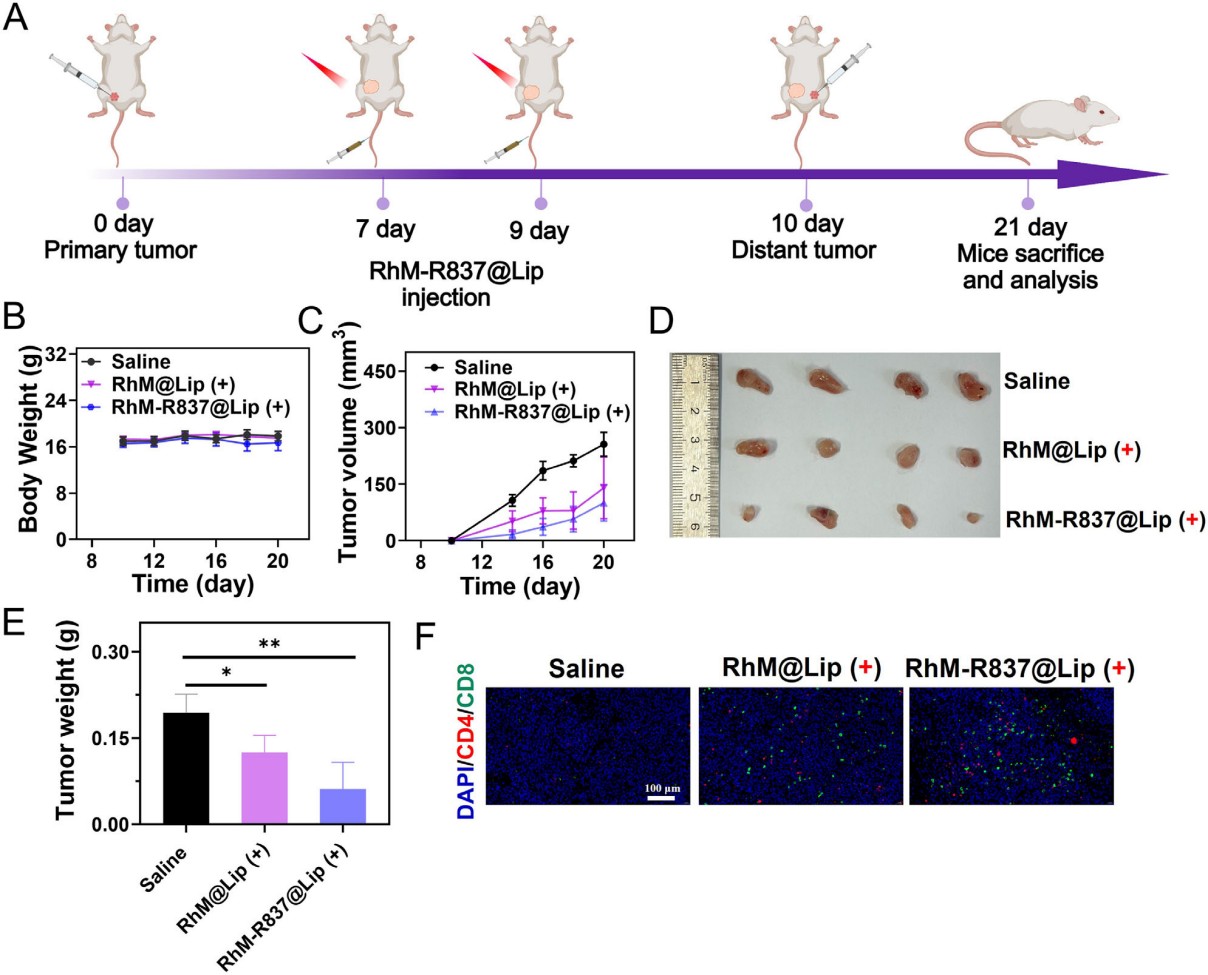
Distal tumor inhibition of **RhM‐R837@Lip**. (A) Schematic diagram of distant tumors evaluation with **RhM‐R837@Lip**. **RhM** dose: 0.5 mg/kg; R837 dose: 1 mg/kg; laser: 808 nm, 0.5 W/cm^2^, 5 min. (B) Body weight measurements of mice in each group during treatment. (C) Distant tumor volume measurements of mice treated with different methods. (D, E) Distant tumor weight and photos of different treatments. (F) Immunofluorescence staining of CD4^+^and CD8^+^ in the tumor sections, scale bars: 100 µm. Blue channel: DAPI stained nuclei (Ex = 359 nm, Em = 457 nm). Green channel: CD8^+^ cells labeled by antibody staining (Ex = 491 nm, Em = 516 nm). Red channel: CD4^+^ cells labeled by antibody staining (Ex = 557 nm, Em = 570 nm). Data are expressed as mean ± SD (n = 4). Statistical significances were calculated via Student's *t* test, *p < 0.05, **p < 0.01 and ***p < 0.001.

To assess the ability of **RhM‐R837@Lip** in suppressing distal tumors and the activation of systemic immune responses, we established distal tumors post‐primary treatment and tracked distal tumor progression. As illustrated in Figure 7A, after two treatments of the left‐side primary tumor, 4T1 cells were implanted into the right mammary fat pad. During the following 11 days, no significant changes in body weight of mice were observed in any group (Figure 7B).

In the control group, distal tumors grew rapidly, with an average volume of 255.9 mm^3^. In contrast, the average volume in the **RhM‐R837@Lip** group was only 100.4 mm^3^, while the **RhM@Lip** showed a moderate volume of 140.2 mm^3^ in (Figure 7C). This indicates that previous phototherapy combined with R837 functioned as a vaccine, triggering a systemic immune response that suppressed the growth of distal tumors. After euthanizing the mice, the tumor weight in the **RhM‐R837@Lip** group was significantly lower than that in the control group, and the tumor suppression effect was superior to that in the **RhM@Lip** group (Figure 7D,E). The suppression of distal tumor growth is predominantly driven by potentiated systemic antitumor immunity. Immunofluorescence analysis revealed significantly enhanced CD4^+^ and CD8^+^ T cell infiltration in distal tumors in the **RhM‐R837@Lip** group compared to controls (Figure 7F). The abscopal effect analysis confirmed that the **RhM‐R837@Lip** combination therapy significantly outperformed phototherapy alone in systemic immune activation.

To further evaluate the in vivo safety profile, we analyzed serum biochemical markers in each treatment group, including liver function markers: alanine aminotransferase (ALT); aspartate aminotransferase (AST); alkaline phosphatase (ALP); γ‐glutamyl transferase (γ‐GT), and kidney function markers: blood urea nitrogen (BUN); creatinine (CR); uric acid (UA). As shown in Figure S37, serum biochemical analysis revealed no clinically significant abnormalities across all treatment groups. H&E staining revealed no apparent damage to major organs (Figure S38). These results confirmed the safety of the systemic administration of **RhM‐R837 @Lip**.

## 3 Conclusion

In summary, we developed a liposomal nanoassembly (**RhM‐R837@Lip**) that integrates Type I photodynamic, photothermal and immunotherapeutic modalities to achieve effective anti‐hypoxia PDT and inhibition of tumor metastasis. In this system, liposomes act as electron donors, enabling excited‐state **RhM** to extract electrons from lipid molecules, thereby enhancing intermolecular electron transfer. This mechanism led to improved Type I photodynamic conversion efficiency and almost complete suppression of ^1^O_2_ generation. Notably, the nanoconfined liposomal architecture also served as an efficient photothermal nanoreactor, achieving a high photothermal conversion efficiency of 56.1%. Furthermore, co‐loading the immunoadjuvant R837 within the liposome successfully activated antitumor immunity, promoting tumor ablation and improving prognosis via systemic immune activation. Compared to **RhM** alone, **RhM‐R837@Lip** exhibited superior photoablation efficacy against solid tumors and more effectively inhibited metastatic progression. Overall, this study presents an innovative nanoplatform strategy for optimizing Type I photosensitizer performance and offers a promising approach for efficient PDT in hypoxic tumor environments.

## 4 Experimental Section

### 4.1 Characterization of RhM‐R837@Lip, R837@Lip, and RhM@Lip

The hydrodynamic particle size and zeta potential of **RhM‐R837@Lip**, **R837@Lip**, and **RhM@Lip** were measured via dynamic light scattering using a Malvern Zetasizer Nano series instrument. Transmission electron microscopy was employed to examine morphology of **RhM‐R837@Lip**, using a 2% uranyl acetate as the negative staining agent. UV–vis absorption spectra were recorded using a Thermo Fisher Evolution 200 UV–vis spectrophotometer. The drug loading capacity (LC) and the encapsulation efficiency (EE) of **RhM** and R837 were determined by UV–vis spectroscopy and high‐performance liquid chromatography (HPLC), respectively. Prior to LC and EC measurement, liposomes were lysed with methanol. The encapsulation efficiency was calculated using the following formula: EE% = (Drug _loaded_ / Drug _added_) × 100%. The drug loading capacity, was calculated as: LC% = (Drug _loaded_ / Total weight of liposomes) ×100%.

### 4.2 Measurement of Photothermal Conversion Efficiency

Photothermal conversion efficiency (PCE) was measured by irradiating **RhM‐R837@Lip** (20 µm in PBS) and gold nanorods (10 µm in PBS) with an 808 nm laser at 0.5 W/cm^2^ for 10 min. After irradiation, the sample was allowed to cool to room temperature, and the temperature was recorded every 30 s. Thermal stability was assessed over four cycles of heating and cooling.

The PCE (η) was calculated using the equation:

$$\eta =\frac{hs\left(T_{\max}-T_{amb}\right)-Q_{dis}}{I\left(1-10^{-A_{808}}\right)},$$

where *h* is the heat transfer coefficient, *s* is the surface area of the container, *Q_dis_* is the heat dissipated by the solvent and container. *I* is the laser power and *A*_808_ is the absorbance at 808 nm. The value *hs* is calculated using:

$$hs=\frac{mC_{solution}}{\tau_{s}},$$

where *m* is the mass of the solution, *C_solution_* is the heat capacity, and *τ_s_* is the time constant obtained from the cooling curve:

$$\tau_{s}=-\frac{t}{\ln\theta },$$

where *t* is the time, *θ* is the dimensionless driving force:

$$\theta =\frac{T-T_{abm}}{T_{\max}-T_{abm}},$$

*T_max_* and *T_amb_* are the maximum system and the ambient temperatures, respectively.

Photothermal conversion efficiencies of **RhM**, **RhM@Lip** and Au nanorods were calculated using the same method.

### 4.3 ROS Detection in Solution

The ROS generation pathway was identified using specific fluorescent probes. Singlet oxygen (^1^O_2_) production was assessed using Singlet Oxygen Sensor Green (SOSG, 5 µm). Sample solutions containing SOSG and **RhM**, **RhM@Lip** or **RhM‐R837@Lip** (**RhM** 10µm ) were irradiated an 808 nm irradiation laser (0.5 W/cm^2^), and the fluorescence intensity at 525 nm was measured at various time intervals.

Hydroxyphenyl fluorescein (HPF, 40 µm) was used to detect hydroxyl radicals (•OH). After 808 nm laser irradiation (0.5 W/cm^2^), fluorescence at 515 nm was recorded in PBS (1 mL) containing HPF and **RhM**, **RhM@Lip**, **or RhM‐R837@Lip** (10 µm each).

Dihydroethidium (DHE, 40 µm) was used to superoxide anion (O_2_^•−^). DHE and **RhM**, **RhM@Lip** or **RhM‐R837@Lip** (10 µm each) were added to 1 mL of water containing calf thymus DNA (ctDNA, 250 µg/mL), followed by irradiation with an 808 nm laser (0.5 W/cm^2^). DHE intercalates into DNA and emits red fluorescence in the presence of O_2_^•−^. Fluorescence spectra were recorded at various time points using a HITACHI F‐7000 fluorescence spectrophotometer.

### 4.4 ESR Analysis

ESR measurements were performed to distinguish different ROS species using DMPO and TEMP as the spin‐trapping agents for O_2_•^−^ and ^1^O_2_, respectively. For DMPO‐based detection, DMPO (10 µL, 0.2 m) was added to the **RhM** solution (50 µL, 0.7 mm) or the **RhM‐R837@Lip** solution (50 µL, 0.7 mm **RhM**), and the characteristic ESR signals were recorded before and after laser irradiation. For TEMP‐based singlet oxygen detection, 10 µL of TEMP was added to the **RhM** solution (100 µL, 0.7 mm) or the **RhM‐R837@Lip** solution (100 µL, 0.7 mm **RhM**), and the corresponding TEMPO adduct signals were monitored upon laser irradiation.

### 4.5 Intracellular ROS Measurement

4T1 cells (1 × 10^4^ cells/dish) were seeded into confocal dishes containing 1 mL of DMEM and incubated for 24 h. The cells were then treated with **RhM@Lip** (0.5 µm, 1 mL), **R837@Lip** or **RhM‐R837@Lip** for 2 h. Afterward, they were stained with 2’,7’‐dichlorodihydrofluorescein diacetate (H_2_DCFH‐DA, 5 µm, 1 mL) for 30 min. Following three PBS washes, the cells were irradiated with 808 nm laser (0.5 W/cm^2^) for 5 min. Fluorescence images were captured using a confocal microscope with an excitation wavelength of 488 nm.

### 4.6 Cellular Uptake

4T1 cells (∼1×10^5^ cells/dish) were seeded in confocal dishes and incubated for 24 h. The cells were then incubated with **RhM‐R837@Lip‐Cy7** (**RhM**: 0.5 µm) for varying durations, cells were washed three times with PBS and observed using a confocal fluorescence microscope.

### 4.7 Cellular Uptake Mechanism Studies

4T1 cells (1 × 10^4^ cells/dish) were seeded in confocal dishes and cultured for 12 h. For the temperature‐dependent uptake studies, cells were incubated with **RhM‐R837@Lip‐Cy5** (0.5 µm) for 6 h at either 37°C or 4°C. For pathway inhibition studies, cells were pretreated for 60 min at 37°C with these inhibitors: Dynasore (25 µg/mL, clathrin‐mediated endocytosis inhibitor), Nystatin (25 µg/mL, caveolin‐mediated inhibitor), and Amiloride hydrochloride (25 µg/mL, macropinocytosis inhibitor). Cells were then incubated with **RhM‐R837@Lip‐Cy5** (0.5 µm) for 6 h at 37°C. After treatment, all cells were washed with PBS and analyzed using a confocal fluorescence microscope and flow cytometry.

### 4.8 Intracellular Colocalization Assay

4T1 cells (1 × 10^4^ cells/dish) were seeded in confocal dishes and incubated for 12 h. Cells were then treated with **RhM‐R837@Lip‐Cy5** (**RhM**: 0.5 µm) for 6 h at 37°C. Afterward, they were stained with Lyso‐Tracker Green (200 nm) or MitoTracker Green (200 nm) for 30 min at 37°C, followed by staining with Hoechst 33342 for 20 min. After three PBS washes, fluorescence images were captured using a confocal microscope.

### 4.9 Cytotoxicity Assays

The cytotoxicity of **RhM‐R837@Lip** was evaluated using the CCK‐8 assay. Briefly, 4T1, Hep G2 and A549 (8 × 10^3^ cells/well) were seeded into 96‐well plates with 100 µL of culture medium per well and incubated overnight at 37°C. The medium was then replaced with fresh medium containing **RhM‐R837@Lip** at various **RhM** concentrations (0‐2 µm), followed by 12 h of incubation. Subsequently, cells were exposed to NIR laser irradiation (808 nm, 0.5 W/cm^2^, 5 min) and further incubated for another 12 h. Afterward, 10 µL of CCK‐8 solution was added to each well and incubated for 2 h. The absorbance at 450 nm was measured using a microplate reader. Cell viability was calculated using the following formula: Cell viability (%) = (OD_Sample_—OD_PBS_) / (OD_Blank_—OD_PBS_) × 100%. For live/dead cell staining, 4T1 cells were first treated with **RhM‐R837@Lip**, **R837@Lip**, and **RhM@Lip** (**RhM**, 0.5 µm) for 12 h. For irradiation groups, cells were then exposed to NIR laser irradiation (808 nm, 0.5 W/cm^2^, 5 min). After 6 h, the cells were co‐stained with calcein acetoxymethyl ester (calcein AM) and propidium iodide (PI) solutions, and fluorescence images were acquired using a confocal fluorescence microscope. Apoptosis was further analyzed using flow cytometry using an Annexin V‐fluorescein isothiocyanate/propidium iodide (Annexin V‐FITC/PI) apoptosis detection kit. Briefly, 4T1 cells were cultured in 12‐well plates and treated with **RhM‐R837@Lip**, **R837@Lip** or **RhM@Lip** (**RhM**, 0.5 µm, 1 mL) for 12 h. For the laser‐treated groups, cells were irradiated with an 808 nm laser (0.5 W/cm^2^, 5 min), followed by 6 h of incubation. Cells were then harvested and stained with Annexin V‐FITC/PI according to the kit protocol, and analyzed using a flow cytometry (BD Biosciences, USA).

### 4.10 Analysis of Mitochondrial Membrane Potential

Mitochondrial membrane potential was assessed using JC‐1 staining. 4T1 cells (8 × 10^4^ cells/dish) were seeded in confocal dishes and incubated for 12 h. Cells were then treated with **RhM‐R837@Lip**, **R837@Lip**, or **RhM@Lip** (**RhM**, 0.5 µm) for 12 h. For laser‐treated groups, cells were irradiated with NIR light (808 nm, 0.5 W/cm^2^, 5 min), followed by a 6 h incubation. Subsequently, cells were stained with JC‐1 (5 µm in PBS) at room temperature for 20 min in the dark. Fluorescent images were obtained using a confocal laser scanning microscopy.

### 4.11 Detection of Adenosine Triphosphate (ATP) Levels

4T1 cells (8 × 10^4^ cells/well) were seeded into 24‐well plates and incubated for 12 h, followed by treatment with RPMI‐1640 medium (containing 10% FBS) supplemented with **RhM‐R837@Lip**, **R837@Lip**, or **RhM@Lip** (**RhM**, 0.5 µm) for 12 h. For laser‐treated groups, cells were irradiated with NIR light (808 nm, 0.5 W/cm^2^, 5 min). After 12 h incubation, the cell supernatants were collected by centrifugation (2000 rpm, 5 min). ATP levels were measured using an ATP Determination Kit (Beyotime, S0026) following the manufacturer's protocol. Luminescence was recorded using a Tecan Spark multimode microplate reader.

### 4.12 Confocal Fluorescence Imaging of CRT and HMGB1

4T1 cells (1×10^6^ cells/dish) were seeded in confocal dishes and cultured overnight. Cells were then treated with **RhM‐R837@Lip**, **R837@Lip**, or **RhM@Lip** (**RhM**, 0.5 µm) at 37°C for 12 h. For the laser‐treated groups, cells were irradiated with NIR light (808 nm, 0.5 W/cm^2^, 5 min), followed by 1 h of incubation. Cells were washed with PBS and fixed with 4% paraformaldehyde for 20 min, then blocked with 10% goat serum for 1 h. For immunostaining, cells were washed three times with PBS and incubated with CRT antibody (Abcam, ab92516) or HMGB1 antibody (Abcam, ab79823) at 4°C for 12 h. After washing, cells were stained with goat anti‐rabbit IgG Alexa Fluor 488 antibody (Abcam, ab150077) in the dark for 1 h at 4°C. Nuclei were counterstained with Hoechst 333421 (µg/mL) for 10 min. Fluorescence images were captured using a Leica SP8 laser confocal microscope.

### 4.13 Detection of HMGB1 by ELISA

4T1 cells (8 × 10^4^ cells/well) were seeded in 24‐well plates and incubated for 12 h, followed by treatment with RPMI‐1640 medium (containing 10% FBS) supplemented with **RhM‐R837@Lip**, **R837@Lip**, or **RhM@Lip** (**RhM**, 0.5 µm) for 12 h. For laser‐treated groups, cells were irradiated with NIR light (808 nm, 0.5 W/cm^2^, 5 min). After an additional 6 h of incubation, the supernatant from each group was collected. HMGB1 concentration was quantified using an ELISA kit (JONLNBIO, JL13702) according to the manufacturer's instructions. Absorbance was measured using a Tecan Spark multimode microplate reader.

### 4.14 In vivo Antitumor Effect of RhM‐R837@Lip

Specific pathogen‐free (SPF) female BALB/c mice (6‐8 weeks) were purchased from Beijing Vital River Laboratory Animal Technology Co., Ltd. All animals were maintained under SPF conditions with a 12 h light/12 h dark cycle and were acclimatized to the animal facility for one week prior to experimentation, with free access to standard chow and water. All animal experiments were approved by the Nankai University Animal Experiment Ethics Committee (2020N221KY) and conducted in accordance with the Guide for the Care and Use of Laboratory Animals of the National Institutes of Health. An orthotopic 4T1 tumor model was established by subcutaneously injecting 4T1 cells (2 × 10^6^ cells per mouse in 100 µL PBS) into the left mammary fat pads of mice. When the tumor volume reached approximately 100 mm^3^, the tumor‐bearing mice were randomly divided into six groups: (i) Saline, (ii) **R837@Lip**, (iii) **RhM@Lip**, (iv) **RhM@Lip** + laser, (v) **RhM‐R837@Lip**, and (vi) **RhM‐R837@Lip** + laser. For the laser irradiation groups, 12 h after tail vein injection of the formulation (**RhM** dose: 0.5 mg/kg; R837 dose: 1 mg/kg), the tumors were irradiated with an 808 nm laser (0.5 W/cm^2^) for 5 min. Tumor temperature changes were monitored using a thermal imaging camera. During the study, images of the mice, tumor volumes, and body weights were recorded every other day. Tumor volume was calculated using the formula: tumor volume = (length × width [2])/2. At the end of experiment, tumor and major organs were harvested and fixed in 4% formalin for histological and immunohistochemical analyses, including H&E, Ki67, PD‐L1, CRT, HMGB1, CD3^+^/CD4^+^, CD3^+^/CD8^+^, CD4^+^/CD25^+^/Foxp3 staining.

### 4.15 DESI‐MSI Analysis of Tumor Tissue

Frozen tumor tissue sections (10 µm) were prepared using a cryostat (Leica CM3600, Germany). The tissue sections were vacuum‐dried for 30 min. Desorption electrospray ionization mass spectrometry imaging (DESI‐MSI) was performed on a Q Exactive UHMR Hybrid Quadrupole‐Orbitrap mass spectrometer (Thermo Scientific, Germany) equipped with a DESI ambient ion source (VikTor, Beijing, China). Analyses were conducted in positive‐ion mode with a mass range of *m/z* 60–900 and a resolving power of 70,000 (FWHM at *m/z* 200). The spray solvent consisted of acetonitrile/water (80:20, *v/v*), delivered at a flow rate of 5 µL/min using a syringe pump (DK Infusetek, Shanghai, China). The electrospray voltage was set to 4.5 kV, and nitrogen was used as the nebulizing gas at a pressure of 0.6 MPa. The spray impact angle was fixed at 60°. Tissue sections were scanned in constant‐velocity mode at 150 µm/s with a pixel size of 50 × 100 µm.

### 4.16 In vivo Anti‐Tumor Immune Response of RhM‐R837@Lip

When the average tumor volume reached approximately 100 mm^3^, the mice were randomly divided into six groups: (i) saline group, (ii) **R837@Lip** group, (iii) **RhM@Lip** group, (iv) **RhM@Lip** + laser group, (v) **RhM‐R837@Lip** group, and (vi) **RhM‐R837@Lip** + laser group. For groups (iv) and (vi), the tumors were irradiated with 808 nm laser (0.5 W/cm^2^, 5 min) 12 h after intravenous injection. One week after treatment, tumors, spleens, and inguinal lymph nodes were harvested to prepare single‐cell suspensions. Tumor and spleen cell suspensions were co‐stained with anti‐CD3‐APC, anti‐CD4‐FITC, anti‐CD8‐PE antibodies following standard protocols, and immediately analyzed by flow cytometry. Cell suspensions from tumor and inguinal lymph nodes were co‐stained with anti‐CD11c‐FITC, anti‐CD80‐PE, and anti‐CD86‐APC antibodies and also analyzed immediately by flow cytometry.

### 4.17 Suppression of Distal Tumor

When the volume of the primary (left‐sided) orthotopic tumor reached approximately 100 mm^3^, the mice were randomly divided into three groups randomly: (i) saline group, (ii) **RhM@Lip** + laser group, and (iii) **RhM‐R837@Lip** + laser group. For the laser irradiation groups, 12 h after tail vein injection of the formulation (**RhM** dose: 0.5 mg/kg, R837 dose: 1 mg/kg), tumors were irradiated with an 808 nm laser (0.5 W/cm^2^) for 5 min. One day after treatment, 4T1 cells (2 × 10^6^ cells per mouse) were inoculated into the right mammary fat to generate a distal tumor. Tumor size on the right side and the body weight were recorded over the following 11 days.

### 4.18 Statistical Analysis

All data were statistically analysed by Prism 8 (Graphpad, USA). All quantitative results are presented as mean ± standard deviation (SD), and the sample size (n) for each experiment is indicated in the corresponding figure captions or text. The data were analyzedusing Student's t tests and 1‐way or 2‐way ANOVA followed by post hoctests. Differences of p <0.05 were considered significant in all statisticalanalyses. Statistically significant differences are shown with asterisks asfollows: *p <0.05, **p <0.01, ***p <0.001, ****p <0.0001, and ns for nosignificance [58].

## Author Contributions

M.Z. conceived the original idea, developed the analytical methodology, and wrote the initial draft of the manuscript. S.L. and M.W. contributed to the methodological refinement and provided critical insights into the experimental design and data analysis. T.Z., J.L, Y.P. and H.F. conducted the experiments and analyzed the data. L.Z., X.H. and M.W performed data visualization, supervised the project, contributed to methodology, and revised the manuscript. L.W. and T.D.J. supervised the project, contributed to methodology, and revised the manuscript. W.Z., B.W. and W.F. contributed to manuscript revision and validation, provided resources and supervision, and oversaw the overall direction of the study. All authors discussed the results and approved the final version of the manuscript.

## Conflicts of Interest

The authors declare no conflicts of interest.

## Supporting information




**Supporting File**: advs74370‐sup‐0001‐SuppMat.docx.

## Data Availability

The data that support the findings of this study are available from the corresponding author upon reasonable request.

## References

[1] M. Overchuk , R. A. Weersink , B. C. Wilson , and G. Zheng , “Photodynamic and Photothermal Therapies: Synergy Opportunities for Nanomedicine,” ACS Nano 17 (2023): 7979–8003, 10.1021/acsnano.3c00891. 37129253 PMC10173698

[2] S. Fu , Z. Chen , L. Li , Y. Wu , Y. Liao , and X. Li , “Redox‐activated Photosensitizers for Visualizing Precise Diagnosis and Potentiating Cancer Therapy,” Coordination Chemistry Reviews 507 (2024): 215734, 10.1016/j.ccr.2024.215734.

[3] H. Li , Y. Kim , H. Jung , J. Y. Hyun , and I. Shin , “Near‐infrared (NIR) Fluorescence‐Emitting Small Organic Molecules for Cancer Imaging and Therapy,” Chemical Society Reviews 51 (2022): 8957–9008, 10.1039/D2CS00722C. 36226744

[4] A. Sharma , P. Verwilst , M. Li , et al., “Theranostic Fluorescent Probes,” Chemical Reviews 124 (2024): 2699–2804, 10.1021/acs.chemrev.3c00778. 38422393 PMC11132561

[5] Y. Wang , K. Ma , M. Kang , et al., “A New Era of Cancer Phototherapy: Mechanisms and Applications,” Chemical Society Reviews 53 (2024): 12014–12042, 10.1039/D4CS00708E. 39494674

[6] Y.‐Y. Zhao , H. Kim , V.‐N. Nguyen , S. Jang , W. J. Jang , and J. Yoon , “Recent Advances and Prospects in Organic Molecule‐Based Phototheranostic Agents for Enhanced Cancer Phototherapy,” Coordination Chemistry Reviews 501 (2024): 215560, 10.1016/j.ccr.2023.215560.

[7] M. Dirak , C. M. Yenici , and S. Kolemen , “Recent Advances in Organelle‐Targeted Organic Photosensitizers for Efficient Photodynamic Therapy,” Coordination Chemistry Reviews 506 (2024): 215710, 10.1016/j.ccr.2024.215710.

[8] J. Gao , Y. Tian , Y. Li , F. Hu , and W. Wu , “Design Strategies for Aggregation‐Induced Emission Photosensitizers With Enhanced Safety in Photodynamic Therapy,” Coordination Chemistry Reviews 507 (2024): 215756, 10.1016/j.ccr.2024.215756.

[9] Z. Fan , S. Wu , H. Deng , G. Li , L. Huang , and H. Liu , “Light‐Triggered Nanozymes Remodel the Tumor Hypoxic and Immunosuppressive Microenvironment for Ferroptosis‐Enhanced Antitumor Immunity,” ACS Nano 18 (2024): 12261–12275, 10.1021/acsnano.4c00844. 38683132

[10] M. Li , J. Xia , R. Tian , et al., “Near‐Infrared Light‐Initiated Molecular Superoxide Radical Generator: Rejuvenating Photodynamic Therapy Against Hypoxic Tumors,” Journal of the American Chemical Society 140 (2018): 14851–14859, 10.1021/jacs.8b08658. 30362735

[11] M. Li , Y. Xu , X. Peng , and J. S. Kim , “From Low to No O_2_‐Dependent Hypoxia Photodynamic Therapy (hPDT): A New Perspective,” Accounts of Chemical Research 55 (2022): 3253–3264, 10.1021/acs.accounts.2c00531. 36323625

[12] T. C. Pham , V.‐N. Nguyen , Y. Choi , S. Lee , and J. Yoon , “Recent Strategies to Develop Innovative Photosensitizers for Enhanced Photodynamic Therapy,” Chemical Reviews 121 (2021): 13454–13619, 10.1021/acs.chemrev.1c00381. 34582186

[13] D. Chen , Q. Xu , W. Wang , J. Shao , W. Huang , and X. Dong , “Type I Photosensitizers Revitalizing Photodynamic Oncotherapy,” Small 17 (2021): 2006742, 10.1002/smll.202006742. 34038611

[14] X. Li , D. Lee , J. D. Huang , and J. Yoon , “Phthalocyanine‐Assembled Nanodots as Photosensitizers for Highly Efficient Type I Photoreactions in Photodynamic Therapy,” Angewandte Chemie International Edition 57 (2018): 9885–9890, 10.1002/anie.201806551. 29927036

[15] Y.‐Y. Wang , Y.‐C. Liu , H. Sun , and D.‐S. Guo , “Type I Photodynamic Therapy by Organic–Inorganic Hybrid Materials: From Strategies to Applications,” Coordination Chemistry Reviews 395 (2019): 46–62, 10.1016/j.ccr.2019.05.016.

[16] T. Xiong , Y. Chen , Q. Peng , et al., “Lipid Droplet Targeting Type I Photosensitizer for Ferroptosis via Lipid Peroxidation Accumulation,” Advanced Materials 36 (2024): 2309711, 10.1002/adma.202309711. 37983647

[17] K. X. Teng , W. K. Chen , L. Y. Niu , W. H. Fang , G. Cui , and Q. Z. Yang , “BODIPY‐Based Photodynamic Agents for Exclusively Generating Superoxide Radical Over Singlet Oxygen,” Angewandte Chemie, International Edition 60 (2021): 19912–19920. 34227724 10.1002/anie.202106748

[18] Z. Ding , Y. Gu , C. Zheng , et al., “Organic Small Molecule‐Based Photothermal Agents for Cancer Therapy: Design Strategies From Single‐Molecule Optimization to Synergistic Enhancement,” Coordination Chemistry Reviews 464 (2022): 214564, 10.1016/j.ccr.2022.214564.

[19] S. Lee , S. Min , G. Kim , and S. Lee , “Recent Advances in the Design of Organic Photothermal Agents for Cancer Treatment: A Review,” Coordination Chemistry Reviews 506 (2024): 215719, 10.1016/j.ccr.2024.215719.

[20] Y. Liu , J. Han , Y. Bo , R. Bhatta , and H. Wang , “Targeted Delivery of Liposomal Chemoimmunotherapy for Cancer Treatment,” Frontiers in Immunology 13 (2022): 1010021, 10.3389/fimmu.2022.1010021. 36341415 PMC9626969

[21] C. Xu and K. Pu , “Second Near‐infrared Photothermal Materials for Combinational Nanotheranostics,” Chemical Society Reviews 50 (2021): 1111–1137, 10.1039/D0CS00664E. 33245316

[22] L. Feng , C. Li , L. Liu , et al., “Acceptor Planarization and Donor Rotation: A Facile Strategy for Realizing Synergistic Cancer Phototherapy via Type I PDT and PTT,” ACS Nano 16 (2022): 4162–4174, 10.1021/acsnano.1c10019. 35230081

[23] Q. Tang , W. Xiao , C. Huang , et al., “Ph‐Triggered and Enhanced Simultaneous Photodynamic and Photothermal Therapy Guided by Photoacoustic and Photothermal Imaging,” Chemistry of Materials 29 (2017): 5216–5224, 10.1021/acs.chemmater.7b01075.

[24] Y. Zhang , M. Zhao , J. Miao , et al., “Hemicyanine‐Based Type I Photosensitizers for Antihypoxic Activatable Photodynamic Therapy,” ACS Materials Letters 5 (2023): 3058–3067, 10.1021/acsmaterialslett.3c00933.

[25] K. Wen , H. Tan , Q. Peng , et al., “Achieving Efficient NIR‐II Type‐I Photosensitizers for Photodynamic/Photothermal Therapy upon Regulating Chalcogen Elements,” Advanced Materials 34 (2022): 2108146, 10.1002/adma.202108146. 34935224

[26] W. Zhang , X. Li , M. Kang , et al., “Anthraquinone‐Centered Type I Photosensitizer With Aggregation‐Induced Emission Characteristics for Tumor‐Targeted Two‐Photon Photodynamic Therapy,” ACS Materials Letters 6 (2024): 2174–2185, 10.1021/acsmaterialslett.4c00600.

[27] Y. Y. Zhao , X. Zhang , Y. Xu , et al., “A Renal Clearable Nano‐Assembly With Förster Resonance Energy Transfer Amplified Superoxide Radical and Heat Generation to Overcome Hypoxia Resistance in Phototherapeutics,” Angewandte Chemie International Edition 63 (2024): 202411514, 10.1002/anie.202411514. 38940633

[28] Y. Zhu , Q. Li , C. Wang , et al., “Rational Design of Biomaterials to Potentiate Cancer Thermal Therapy,” Chemical Reviews 123 (2023): 7326–7378, 10.1021/acs.chemrev.2c00822. 36912061

[29] G. Feng , G. Q. Zhang , and D. Ding , “Design of Superior Phototheranostic Agents Guided by Jablonski Diagrams,” Chemical Society Reviews 49 (2020): 8179–8234, 10.1039/D0CS00671H. 33196726

[30] C. Wang , P. Zhao , D. Jiang , et al., “In Situ Catalytic Reaction for Solving the Aggregation of Hydrophobic Photosensitizers in Tumor,” ACS Applied Materials & Interfaces 12 (2020): 5624–5632, 10.1021/acsami.9b21589. 31918542

[31] W. Chen , Z. Wang , M. Tian , et al., “Integration of TADF Photosensitizer as “Electron Pump” and BSA as “Electron Reservoir” for Boosting Type I Photodynamic Therapy,” Journal of the American Chemical Society 145 (2023): 8130–8140, 10.1021/jacs.3c01042. 37001012

[32] Y. Yang , Y. Wang , Y. Liu , et al., “Tumor Oxygen Microenvironment‐Tailored Electron Transfer‐Type Photosensitizers for Precise Cancer Therapy,” Chemical Science 15 (2024): 17032–17040, 10.1039/D4SC03424D. 39328193 PMC11421038

[33] J. Qu , Y. Zhang , Z. Cai , et al., “An Acceptor‐Shielding Strategy of Photosensitizers for Enhancing the Generation Efficiency of Type I Reactive Oxygen Species and the Related Photodynamic Immunotherapy,” Nanoscale 14 (2022): 14064–14072, 10.1039/D2NR02273G. 36053244

[34] T. Xiong , Y. Chen , Q. Peng , et al., “Heterodimeric Photosensitizer as Radical Generators to Promoting Type I Photodynamic Conversion for Hypoxic Tumor Therapy,” Advanced Materials 37 (2025): 2410992, 10.1002/adma.202410992. 39865773

[35] J. Zhuang , S. Liu , B. Li , et al., “Electron Transfer Mediator Modulates Type II Porphyrin‐Based Metal–Organic Framework Photosensitizers for Type I Photodynamic Therapy,” Angewandte Chemie International Edition 137 (2024): 202420643. 10.1002/anie.202420643 39560938

[36] J. Zhuang , G. Qi , Y. Feng , et al., “Thymoquinone as an Electron Transfer Mediator to Convert Type II Photosensitizers to Type I Photosensitizers,” Nature Communications 15 (2024): 4943, 10.1038/s41467-024-49311-z. PMC11164902 38858372

[37] J. Hua , P. Wu , L. Gan , et al., “Current Strategies for Tumor Photodynamic Therapy Combined With Immunotherapy,” Frontiers in Oncology 11 (2021): 738323, 10.3389/fonc.2021.738323. 34868932 PMC8635494

[38] C. W. Ng , J. Li , and K. Pu , “Recent Progresses in Phototherapy‐Synergized Cancer Immunotherapy,” Advanced Functional Materials 28 (2018): 1804688, 10.1002/adfm.201804688.

[39] L. Zhao , S. Zhuo , T. Wang , et al., “Cyclic Dinucleotide Self‐Assembled Nanoparticles as a Carrier‐Free Delivery Platform for STING‐Mediated Cancer Immunotherapy,” CCS Chemistry 6 (2024): 177–195, 10.31635/ccschem.023.202302751.

[40] Z. Chen , W. Guo , L. Tan , et al., “Biomimetic MOF‐Based Nano‐Immunoactivator via Disruption of Ion Homeostasis for Strengthened Tumor Microwave‐Immunotherapy,” Advanced Functional Materials 34 (2024): 2401359, 10.1002/adfm.202401359.

[41] C. Li , L. Tu , Y. Xu , et al., “A NIR‐Light‐Activated and Lysosomal‐Targeted Pt(II) Metallacycle for Highly Potent Evoking of Immunogenic Cell Death that Potentiates Cancer Immunotherapy of Deep‐Seated Tumors,” Angewandte Chemie International Edition 63 (2024): 202406392, 10.1002/anie.202406392. 38775364

[42] Y. Lu , Y. Wang , W. Liu , et al., “Photothermal “Nano‐Dot” reactivate “Immune‐Hot” for Tumor Treatment Via Reprogramming Cancer Cells Metabolism,” Biomaterials 296 (2023): 122089, 10.1016/j.biomaterials.2023.122089. 36898223

[43] C. Chen , X. Ni , S. Jia , et al., “Massively Evoking Immunogenic Cell Death by Focused Mitochondrial Oxidative Stress using an AIE Luminogen With a Twisted Molecular Structure,” Advanced Materials 31 (2019): 1904914, 10.1002/adma.201904914. 31696981

[44] X. Jiang , J. Liu , M. J. Lee , et al., “Nanoscale Coordination Polymer Synergizes Photodynamic Therapy and Toll‐Like Receptor Activation for Enhanced Antigen Presentation and Antitumor Immunity,” Biomaterials 302 (2023): 122334, 10.1016/j.biomaterials.2023.122334. 37776767 PMC10841466

[45] Q. Chen , L. Xu , C. Liang , C. Wang , R. Peng , and Z. Liu , “Photothermal Therapy With Immune‐Adjuvant Nanoparticles Together With Checkpoint Blockade for Effective Cancer Immunotherapy,” Nature Communications 7 (2016): 13193, 10.1038/ncomms13193. PMC5078754 27767031

[46] C. Song , H. Phuengkham , Y. S. Kim , et al., “Syringeable Immunotherapeutic Nanogel Reshapes Tumor Microenvironment and Prevents Tumor Metastasis and Recurrence,” Nature Communications 10 (2019): 3745, 10.1038/s41467-019-11730-8. PMC6702226 31431623

[47] X. Wang , Z. Shi , J. Luo , et al., “Ultrasound Improved Immune Adjuvant Delivery to Induce DC Maturation and T Cell Activation,” Journal of Controlled Release 349 (2022): 18–31, 10.1016/j.jconrel.2022.06.054. 35780954

[48] J. Yue , Q. Mei , P. Wang , P. Miao , W.‐F. Dong , and L. Li , “Light‐Triggered Multifunctional Nanoplatform for Efficient Cancer Photo‐Immunotherapy,” Journal of Nanobiotechnology 20 (2022): 181, 10.1186/s12951-022-01388-8. 35392911 PMC8991811

[49] M. Zhang , S. Wang , Y. Bai , et al., “A Dual‐Function Hemicyanine Material With Highly Efficient Photothermal and Photodynamic Effect Used for Tumor Therapy,” Advanced Healthcare Materials 13 (2023): 2303432, 10.1002/adhm.202303432. 38069831

[50] X. Zhang , K. M. Barraza , K. T. Upton , and J. L. Beauchamp , “Subtle Changes in Lipid Environment Have Profound Effects on Membrane Oxidation Chemistry,” Journal of the American Chemical Society 140 (2018): 17492–17498, 10.1021/jacs.8b08610. 30461271

[51] L. Jiao , Y. Liu , X. Zhang , et al., “Constructing a Local Hydrophobic Cage in Dye‐Doped Fluorescent Silica Nanoparticles to Enhance the Photophysical Properties,” ACS Central Science 6 (2020): 747–759, 10.1021/acscentsci.0c00071. 32490191 PMC7256957

[52] J. Rak , M. Kabesova , and J. Benes , “Advances in Liposome‐Encapsulated Phthalocyanines for Photodynamic Therapy,” Life 13 (2023): 305, 10.3390/life13020305. 36836662 PMC9965606

[53] T. B. Gandek , L. van der Koog , and A. Nagelkerke , “A Comparison of Cellular Uptake Mechanisms, Delivery Efficacy, and Intracellular Fate Between Liposomes and Extracellular Vesicles,” Advanced Healthcare Materials 12 (2023): 2300319, 10.1002/adhm.202300319. 37384827 PMC11469107

[54] K. Un , K. Sakai‐Kato , Y. Oshima , T. Kawanishi , and H. Okuda , “Intracellular Trafficking Mechanism, From Intracellular Uptake to Extracellular Efflux, for Phospholipid/Cholesterol Liposomes,” Biomaterials 33 (2012): 8131–8141, 10.1016/j.biomaterials.2012.07.030. 22858002

[55] B. Ding , P. Zheng , J. Tan , et al., “Sodium Bicarbonate Nanoparticles for Amplified Cancer Immunotherapy by Inducing Pyroptosis and Regulating Lactic Acid Metabolism,” Angewandte Chemie International Edition 62 (2023): 202307706, 10.1002/anie.202307706. 37587061

[56] Y. Ma , Y. Zhang , X. Li , et al., “Near‐Infrared II Phototherapy Induces Deep Tissue Immunogenic Cell Death and Potentiates Cancer Immunotherapy,” ACS Nano 13 (2019): 11967–11980, 10.1021/acsnano.9b06040. 31553168

[57] X. Tang , X. Chen , S. Zhang , et al., “Silk‐Inspired In Situ Hydrogel With Anti‐Tumor Immunity Enhanced Photodynamic Therapy for Melanoma and Infected Wound Healing,” Advanced Functional Materials 31 (2021): 2101320, 10.1002/adfm.202101320.

[58] D. Liu , L. Fu , L. Gong , et al., “Proton‐Gradient‐Driven Porphyrin‐Based Liposome Remote‐Loaded With Imiquimod as In Situ Nanoadjuvants for Synergistically Augmented Tumor Photoimmunotherapy,” ACS Applied Materials & Interfaces 16 (2024): 8403–8416, 10.1021/acsami.3c17133. 38334116

