# Supplementary material for: Liposomal Nanoconfinement Enables Type I Photodynamic Conversion for Synergistic Cancer Photothermal‐Immunotherapy
Source: Adv Sci (Weinh). 2026 Feb 15;13(23):e15013. doi: 10.1002/advs.202515013 (PMC13104119; doi:10.1002/advs.202515013)
Supplement: Supplementary file 1 — Supporting File: advs74370‐sup‐0001‐SuppMat.docx. [file ADVS-13-e15013-s001.docx]

*Supporting Information*

**Liposomal Nanoconfinement Enables Type I Photodynamic Conversion for Synergistic Cancer Photothermal-Immunotherapy**

Minglu Zhang,^a,b,c^ Shanshan Liang,^b^ Meng Wang,^b,d^ Tingfeng Zhang,^b,d^ Jinke Liu,^b^ Yuyuan Peng,^b^ Hao Fang,^b,d^ Lingna Zheng,^b^ Xiao He,^b^ Meng Wang,^b^ Luling Wu,^e,f^* Tony D. James,^f^ Wei Zhao,^a,c^* Bing Wang,^b^* and Weiyue Feng^b^

^a^Qingdao Central Hospital, NHC Key Laboratory of Cardiopulmonary Rehabilitation and Functional Recovery, Industry-Academia-Research Collaborative and Innovation Center for Intelligent Rehabilitation Drug R&D of Shandong Province, Qingdao Key Laboratory of Precision Drug Research for Chronic Disease Rehabilitation, School of Health and Life Sciences, University of Health and Rehabilitation Sciences, Qingdao 266113, China

^b^CAS Key Laboratory for Biomedical Effects of Nanomaterials and Nanosafety, Institute of High Energy Physics, Chinese Academy of Sciences, Beijing, 100049, China

^c^State Key Laboratory of Medicinal Chemical Biology, College of Pharmacy, Key Laboratory of Molecular Drug Research and KLMDASR of Tianjin, Nankai University, Tongyan Road, Haihe Education Park, Tianjin 300350, China

^d^University of Chinese Academy of Sciences, Beijing 100049, China

^e^State Key Laboratory of Analytical Chemistry for Life Science, School of Chemistry, Nanjing University, 163 Xianlin Avenue, Nanjing, 210023, China

^f^Department of Chemistry, University of Bath, Bath BA2 7AY, U.K.

***Corresponding author:**

Luling Wu

E-mail: wllcyl@126.com

Wei Zhao

E-mail: zhaowei1@uhrs.edu.cn

Bing Wang

E-mail: wangbing@ihep.ac.cn

**Experimental Section**

**1 Materials and Instruments**

All chemical reagents were obtained from commercial suppliers and used without further purification. 1,2-Dioleoyl-sn-glycero-3-phosphocholine (DOPC; cat. no.850375) and 1,2-distearoyl-sn-glycero-3-phosphoethanolamine-N-[methoxy(polyethylene glycol)-2000] (DSPE-PEG_2k_; cat. no. 880120) were purchased from Avanti Polar Lipids (USA). DSPE-PEG_2k_-Cyanine7 (DSPE-PEG_2k_-Cy7; cat. no. 80031902) and DSPE-PEG_2k_-Cyanine5 (DSPE-PEG_2k_-Cy5; cat. no.80031707) were obtained from Tanshtech Co., Ltd (Guangzhou, China). Cholesterol and imiquimod (R837) were supplied from Aladdin Biochemical Technology Co., Ltd. (Shanghai, China). Dimethyl sulfoxide (DMSO), high-glucose Dulbecco’s modified Eagle’s medium (DMEM), RPMI 1640 medium, Annexin V-FITC/PI apoptosis detection kit, and Calcein AM/PI assay kit were purchased from Solarbio Biotech (Beijing, China). 2’,7’-Dichlorodihydrofluorescein diacetate (DCFH-DA) and Cell Counting Kit-8 (CCK-8) were purchased from Meilun Biotech (Dalian, China). Hydroxyphenyl fluorescein (HPF), dihydroethidium (DHE), 1,3-diphenylisobenzofuran (DPBF), Singlet Oxygen Sensor Green (SOSG), LysoTracker Green (LTG), MitoTracker Green (MTG), Hoechst 33342, JC-1 mitochondrial membrane potential assay kit, and ATP assay kit were purchased from Beyotime Biotechnology Co., Ltd (Shanghai, China). CRT and HMGB1 antibodies were obtained from Abcam (Shanghai, China). Female BALB/c mice (16-20 g) were obtained from Beijing Vital River Laboratory Animal Breeding Co., Ltd (Beijing, China). All animal experiments were approved by the Nankai University Animal Experiment Ethics Committee (2020N221KY) and conducted in accordance with the Guide for the Care and Use of Laboratory Animals of the National Institutes of Health.

UV-vis absorption spectra were recorded using a Thermo Fisher Evolution 200 spectrometer. Fluorescence spectra were measured on a HITACHI F-7000 fluorescence spectrophotometer. ^1^H and ^13^C NMR spectra were obtained using Bruker AVANCE 400 NMR spectrometer (400 MHz for ^1^H and 100 MHz for ^13^C). High-resolution mass spectrometry (HRMS) data were collected on a Shimadzu ion trap time-of-flight mass spectrometer (MS-IT-TOF, Japan). CCK-8 assays were performed using a Tecan Spark multimode microplate reader. Flow cytometry was conducted using a BD Biosciences flow cytometer (USA). Cellular fluorescence images were captured using a Leica TCS SP8 confocal laser scanning microscope (Germany). *In vivo* fluorescence imaging was carried out using a NightOWL Ⅱ LB983 imaging system (Germany).

**2 Synthetic Procedures**

**Scheme S1.** Synthetic route of **RhM**.

**2.1 Synthesis of** **2-[6-(diethylamino)-4-formyl-2,3-dihydro-1H-xanthen-9-yl]benzoic acid**

Cyclohexanone (3.3 g, 33.5 mmol) and 2-[4-(diethylamino)-2-hydroxybenzoyl]benzoic acid (5.0 g, 16 mmol) were added to concentrated H_2_SO_4_ (25 mL) and stirred at 90 °C for 3 h. After cooling to room temperature, the reaction mixture was slowly poured into crushed ice, followed by addition of HClO_4_ (3.5 mL). The resulting precipitate was filtered, dissolved in DMF (15 mL), and cooled to 0 °C before the dropwise addition of POCl_3_ (5 mL). The reaction mixture was stirred at room temperature for 1 h, then poured into ice water and extracted with CH_2_Cl_2_. The organic layer was concentrated under reduced pressure, dissolved with MeOH (3 mL), and added to saturated NaOH solution. After neutralization with 1 M HCl, the solution was extracted with CH_2_Cl_2_ and purified by silica gel column chromatography (EA:PE = 4:1) to afford the desired product as an orange-yellow solid (2.35 g, 5.8 mmol, yield 36%). ^1^H NMR (400 MHz, CDCl_3_) δ 10.20 (s, 1H), 8.13 (d, *J* = 7.8 Hz, 1H), 7.60 (dd, *J* = 7.6 Hz, 1H), 7.49 (dd, *J* = 7.6 Hz, 1H), 7.13 (d, *J* = 7.6 Hz, 1H), 6.41 (d, *J* = 8.87 Hz, 1H), 6.39 (d, *J* = 2.33 Hz, 1H), 6.25 (dd, *J* = 8.9, 2.5 Hz, 1H), 3.33 (q, *J* = 7.1 Hz, 4H), 2.39 -2.35 (m, 2H), 2.13(t, *J* = 7.1 Hz, 2H), 1.56 (m, 2H), 1.14 (t, *J* = 7.0 Hz, 6H). ^13^C NMR (100 MHz, CDCl_3_) δ 187.23, 167.99, 163.17, 153.65, 149.45, 141.50, 137.18, 132.60, 131.19, 130.29, 130.05, 128.31, 126.70, 119.32, 111.62, 111.23, 107.86, 96.93, 44.57, 27.33, 21.39, 20.40, 12.52.

**2.2 Synthesis of Rh-COOH**

2-[6-(Diethylamino)-4-formyl-2,3-dihydro-1H-xanthen-9-yl]benzoic acid (2.4 g, 5.8 mmol) and K_2_CO_3_ (1.6 g, 11.6 mmol) was added to acetic anhydride (20 mL), followed by the addition of 1-ethyl-2-methyl-4-benzo[c, d]indole (1.4 g, 6.9 mmol). The mixture was stirred at 80℃ for 3 h and then evaporated under reduced pressure. The residue was diluted with CH_2_Cl_2_, washed with saturated aqueous KI solution (3×100 mL), dried over Na_2_SO_4_ and purified by silica gel chromatography (DCM/MeOH = 30:1) to afford **Rh-COOH** as a brown solid (2.73 g, 3.9 mmol, yield 66.4%). ^1^H NMR (400 MHz, DMSO-*d*_6_) δ 8.65 (d, *J* = 13.6 Hz, 1H), 8.33 (d, *J* = 8 Hz, 1H), 8.20 (d, *J* = 7.4 Hz, 1H), 8.09 (d, *J* = 7.9 Hz, 1H), 7.85 - 7.78 (m, 3H), 7.57 - 7.51 (m , 2H), 7.37 - 7.34 (m, 2H), 7.00 (dd, *J* = 7.4, 1.7 Hz, 1H), 6.90 (s, 1H), 6.77 (d, *J* = 8 Hz, 1H), 6.47 (d, *J* = 13.6 Hz, 1H), 4.25 (q, *J* = 7.1 Hz, 2H), 3.60 (q, *J* = 7.6 Hz, 4H), 2.71(t, *J* = 7.0 Hz, 2H), 2.38 - 2.21 (m, 2H), 1.82 - 1.67 (m, 2H), 1.33 (t, *J* = 7.1 Hz, 3H), 1.25 (t, *J* = 7.0 Hz, 6H). ^13^C NMR (100 MHz, DMSO-*d*_6_) δ 167.14, 162.50, 156.37, 155.11, 152.97, 152.12, 141.31, 138.38, 135.34, 133.45, 131.36, 130.61, 130.46, 130.22, 130.22, 130.19, 130.16, 129.95, 129.81, 128.71, 127.56, 125.47, 121.63, 121.63, 120.02, 114.54, 108.26, 104.17, 96.34, 45.27, 38.61, 26.59, 24.30, 20.60, 13.99, 12.94. HRMS (ESI+): calculated [M-I]^+^ 581.2799 m/z, found 581.2798 m/z. M represents C_39_H_37_N_2_O_3_I (chemical formula of compound Rh-COOH).

**2.3 Synthesis of RhM**

Rh-COOH (0.5 g, 0.71 mmol) was dissolved in MeOH (50 mL), followed by the addition of concentrated H_2_SO_4_ (5 mL). The mixture was stirred at 60 °C for 24 h. After cooling, the solvent was partially evaporated, and the residue was slowly poured into crushed ice. The resulting solution was neutralized with 1 M NaOH, extracted with CH_2_Cl_2_, and purified by silica gel column chromatography (DCM/MeOH = 40:1) to afford **RhM** as a brown solid (0.257 g, 0.35 mmol, yield 49.3%). ^1^HNMR (400 MHz, DMSO-*d*_6_) δ 8.85 (d, *J* = 13.7 Hz, 1H), 8.49 (d, *J* = 7.4 Hz, 1H), 8.20 (dd, *J* = 8.1, 1.3 Hz, 1H), 8.17 (d, *J* = 8.0 Hz, 1H), 7.88 (dt, *J* = 14.8, 7.6 Hz, 2H), 7.77 (t, *J* = 7.7 Hz, 1H), 7.65 (d, *J* = 8.2 Hz, 1H), 7.62 – 7.58 (m, 1H), 7.42 (dd, *J* = 10.5, 7.3 Hz, 2H), 7.02 (s, 1H), 6.98 (dd, *J* = 9.3, 2.5 Hz, 1H), 6.74 (d, *J* = 9.2 Hz, 1H), 6.60 (d, *J* = 13.8 Hz, 1H), 4.33 (d, *J* = 5.8 Hz, 2H), 3.70 (s, 3H), 3.61 (q, *J* = 7.4 Hz, 4H), 2.78 (d, *J* = 6.8 Hz, 2H), 2.35 - 2.28 (m, 1H), 2.23 (d, *J* = 6.4 Hz, 1H), 1.76 (td, *J* = 16.6, 6.1 Hz, 2H), 1.36 (t, *J* = 7.1 Hz, 3H), 1.24 (t, *J* = 7.1 Hz, 6H). ^13^C NMR (100 MHz, DMSO-*d*_6_) δ 165.91, 162.64, 156.45, 154.14, 152.98, 152.47, 141.32, 138.91, 135.49, 133.95, 131.16, 130.67, 130.34, 130.25, 130.06, 129.20, 128.56, 125.45, 121.86, 121.69, 120.12, 114.51, 114.36, 108.57, 104.47, 96.52, 52.94, 45.25, 38.69, 26.57, 24.38, 20.61, 14.00, 12.93. HRMS (ESI+): calculated [M-I]^+^ 595.2955 m/z, found 595.2954 m/z. M represents C_40_H_39_N_2_O_3_I (chemical formula of compound RhM).

**3 Preparation of RhM-R837@Lip**

**Scheme S2.** Schematic illustration of the preparation of **RhM-R837@Lip**.

**RhM@Lip** was prepared following a previously reported method.^1^ Briefly, DOPC, cholesterol, DSPE-PEG_2k_, and **RhM** (in a mass ratio of 12:3:4.5:0.25) were dissolved in chloroform. A thin lipid film was formed in a round-bottom flask by evaporating the organic solvent under reduced pressure at 50 °C, followed by vacuum drying for 5 hours. The resulting lipid film was rehydrated with 300 mM citric acid at 60 °C for 20 minutes to form vesicles, which were further dispersed by sonication. The vesicles were then sequentially extruded sequentially through 200 nm and 100 nm membrane polycarbonate membranes to ensure uniform size distribution, followed by purification *via* dialysis. To establish a proton gradient, the lipid suspension was passed through a Sephadex G-25 column equilibrated with PBS (pH 7.4), yielding **RhM@Lip**. Subsequently, **R837** (lipids/drug = 20 mg:1 mg) was loaded into the **RhM@Lip** by incubation at 40 °C for 1 hour. Unencapsulated drug was removed *via* dialysis, resulting in the final **RhM-R837@Lip** formulation. **R837@Lip** was prepared using the same procedure, but starting with blank liposomes prior to **R837** loading.

**Scheme S3.** Schematic illustration of the preparation of **RhM-R837@Lip-Cy5**.

The synthesis strategy for **RhM-R837@Lip-Cy5** is illustrated in Scheme S3. In this formulation, DSPE-PEG_2k_ was replaced with DSPE-PEG_2k_-Cy5, while all other synthetic steps were identical to those used for **RhM-R837@Lip**. Similarly, **RhM-R837@Lip-Cy7** was prepared by replacing DSPE-PEG_2k_ with DSPE-PEG_2k_-Cy7, with the rest of the procedure remaining unchanged.

**Figure S1**. ^1^H NMR spectrum of 2-[6-(diethylamino)-4-formyl-2,3-dihydro-1*H*-xanthen-9-yl]benzoic acid in CDCl_3_.

**Figure S2**. ^13^C NMR spectrum of 2-[6-(diethylamino)-4-formyl-2,3-dihydro-1*H*-xanthen-9-yl]benzoic acid in CDCl_3_.

**Figure S3**. ^1^H NMR spectrum of **Rh-COOH** in DMSO-*d_6_*.

**Figure S4**. ^13^C NMR spectrum of **Rh-COOH** in DMSO-*d_6_*.

**Figure S5**. HRMS spectrum of **Rh-COOH**.

**Figure S6**. ^1^H NMR spectrum of **RhM** in DMSO-*d_6_*.

**Figure S7**. ^13^C NMR spectrum of **RhM** in DMSO-*d_6_*.

**Figure S8**. HRMS spectrum of **RhM**.

**Figure S9.** Characterization of **RhM-R837@Lip**. (A) Dynamic light scattering analysis of **RhM@Lip**, **R837@Lip**, and **RhM-R837@Lip** in aqueous solution. (B) Photographs of **RhM@Lip**, **R837@Lip**, and **RhM-R837@Lip** in aqueous solution. (C) Zeta potentials of **RhM@Lip**, **R837@Lip**, and **RhM-R837@Lip**.

**Figure S10.** The encapsulation efficiency and drug loading of R837 evaluated by HPLC. (A) HPLC analysis of free R837 (25 μM-200 μM). (B) Standard curve of free R837 based on HPLC absorption peak at 325 nm. (C) HPLC analysis of **R837@Lip** and **RhM-R837@Lip** after methanol lysis.

**Figure S11**. Dynamic light scattering analysis of free **RhM** in PBS.

**Figure S12**. The absorption spectra of **RhM-R837@Lip** and **RhM**. (A) Absorption spectra of **RhM-R837@Lip** in PBS with gradually increasing concentrations from 1.25 Μm to 40 Μm. (B) Relationship between the absorbance at 834 nm in (A) and the concentration of **RhM-R837@Lip** in PBS. (C) Absorption spectra of **RhM** in PBS with gradually increasing concentrations from 5 μM to 80 μM. (D) Relationship between the absorbance at 730 nm in (C) and the concentration of **RhM** in PBS. (E) Molar extinction coefficients of of **RhM-R837@Lip** and **RhM**.

**Figure S13**. Infrared thermal images of **RhM@Lip** (20 μM) under 808 nm laser irradiation (808 nm, 1 W/cm^2^) at different time points (0-8 min).

**Figure S14**. Evaluation of photothermal conversion efficiency using the Roper method. (A) Temperature variation of **RhM@Lip** during on–off laser irradiation cycle (808 nm, 1 W/cm^2^). (B) Plot of time versus −ln(θ) for **RhM@Lip**, where θ represents driving force of heat transfer. (C) Temperature variation of **RhM-R837@Lip** during on–off laser irradiation (808 nm, 1 W/cm^2^). (D) UV–Vis absorption spectrum of Au nanorods (10 μM). (E) Temperature variation of **RhM** during on–off laser irradiation (808 nm, 1 W/cm^2^). (F) Plot of time versus −ln(θ) for **RhM**. (G) Temperature variation of Au nanorods during the laser on–off cycle (808 nm, 1 W/cm^2^). (H) Plot of time versus −ln(θ) of Au nanorods.

**Figure S15**. Photostability of **RhM-R837@Lip**. (A) UV–Vis absorption spectra of **RhM-R837@Lip** after 808 nm laser irradiation (1W/cm^2^) for 10 min. (B) Particle size evolution of **RhM-R837@Lip** under light irradiation measured by dynamic light scattering. (C) Particle size distribution of **RhM-R837@Lip** before and after irradiation.

**Figure S16.** *In vitro* photodynamic properties. (A) Singlet Oxygen Sensor Green (SOSG, 5 μM) was employed as a fluorescent probe to monitor ^1^O_2_ generation of **RhM,** **RhM@Lip** and **RhM-R837@Lip** (RhM 10 μM, R837:RhM mass ratio = 2:1) under 808 nm irradiation (0.5 W/cm^2^), Ex: 504 nm, Em: 510-650 nm. (B) •OH generation by **RhM,** **RhM@Lip** and **RhM-R837@Lip** (RhM 10 μM, R837:RhM mass ratio = 2:1) detected using HPF (40 μM) as the fluorescent probe, Ex: 490 nm, Em: 500 nm-650 nm. (C) O_2_^•−^ generation by **RhM,** **RhM@Lip** and **RhM-R837@Lip** (RhM 10 μM, R837:RhM mass ratio = 2:1) detected using DHE (40 μM) as the fluorescent probe, Ex: 490 nm, Em: 500 nm-750 nm.

**Figure S17**. Time-dependent DPBF decay of **RhM**, **RhM-R837@Lip** and ICG under 808 nm irradiation (1 W/cm^2^) within 7 min.

**Figure S18**. Transient absorption spectra of **RhM-R837@Lip** in PBS. (A) Transient absorption spectra of **RhM-R837@Lip** excited with a 730 nm laser. (B) Corresponding kinetic trace at 622 nm. (C) Transient absorption spectra of **RhM-R837@Lip** excited with a 730 nm laser. (D) Corresponding kinetic curve at 835 nm.

**Figure S19**. Transient absorption spectra of **RhM** in PBS. (A) Transient absorption spectra of **RhM** excited with a 730 nm laser. (B) Corresponding kinetic trace at 622 nm. (C) Corresponding kinetic trace at 675 nm. (D) Transient absorption spectra of **RhM** excited with a 730 nm laser. (E) Corresponding kinetic trace at 839 nm. (F) Corresponding kinetic trace at 1090 nm.

**Figure S20**. *In vitro* photodynamic properties of Hypocrellin B and Hypocrellin B@Lip. (A) •OH generation generation upon irradiation (0.5 W/cm^2^) of Hypocrellin B and Hypocrellin B@Lip (5 μM), detected using HPF (40 μM) as the fluorescent probe (Ex: 490 nm, Em: 500-650 nm). (B) O_2_^•−^ generation upon irradiation (0.5 W/cm^2^) of Hypocrellin B and Hypocrellin B@Lip (5 μM), detected using DHE (40 μM) as the fluorescent probe (Ex: 490 nm, Em: 500-750 nm).

**Figure S21**. Confocal fluorescence images of 4T1 cells incubated with **RhM-R837@Lip-Cy7** (**RhM** 0.5 μM, mass ratio of R837 to RhM = 2:1) for different durations. Blue channel: Ex = 405 nm, Em = 430-475 nm. Red channel: Ex = 640 nm, Em = 663-738 nm. Scale bar: 60 μm.

**Figure S22.** Cytotoxicity analysis of **ICG**, **Chlorin e6**, and **RhM-R837@Lip** in 4T1cells under 808 nm irradiation (0.5 W/cm^2^, 5 min).

**Figure S23**. Cytotoxicity analysis of **R837@Lip**, **RhM@Lip**, and **RhM-R837@Lip** in HUVEC and CHL cells under dark conditions. (A) Cell viability of HUVEC treated with various concentrations of **R837@Lip**, **RhM@Lip** and **RhM-R837@Lip** (RhM: 0-2 μM, with a mass ratio of R837 to RhM = 2:1) at 37 °C for 24 h in the dark. (B) Cell viability of CHL cells treated with various concentrations of **R837@Lip**, **RhM@Lip** and **RhM-R837@Lip** (RhM 0-2 μM, with a mass ratio of R837 to RhM = 2:1) at 37 °C for 24 h in the dark.

**Figure S24**. Calcein-AM and PI co-staining analysis of **RhM-R837@Lip**. (A) Calcein-AM and PI co-staining in 4T1 cells under different treatment conditions. Scale bar: 200 μm. Green channel: Ex=488 nm, Em = 500-550 nm. (B) Quantitative analysis of red and green fluorescence in different groups from (A). Experimental conditions: **RhM** 0.5 μM, R837: **RhM** = 2:1 (mass ratio), (+) indicates laser irradiation (808 nm, 0.5 W/cm², 5 min). Data are expressed as mean ± SD (n = 3). Statistical significance was determined by Student’s *t-*test: *p < 0.05, **p < 0.01, ***p < 0.001.

**Figure S25**. Mitochondrial membrane potential analysis of **RhM-R837@Lip**. (A) Mitochondrial membrane potential in 4T1 cells under different treatments, detected by confocal laser scanning microscopy. Scale bar: 50 μm. Green channel: Ex=488 nm, Em = 500-550 nm. Red channel: Ex = 640 nm, Em = 663-738 nm. (B) Quantitative analysis of red and green fluorescence intensities in different groups. Experimental conditions: RhM 0.5 μM, R837: RhM = 2:1 (mass ratio), (+) indicates laser irradiation (808 nm, 0.5 W/cm², 5 min). Data are presented as mean ± SD (n = 3). Statistical significance was determined by Student’s *t*-test: *p < 0.05, **p < 0.01, ***p < 0.001.

**Figure S26**. Confocal fluorescence images of 4T1 tumor sections at 12 h post tail-vein injection of **RhM-R837@Lip-Cy7**. Blue channel: Ex = 405 nm, Em = 430-475 nm. Red channel: Ex = 640 nm, Em = 663-738 nm. (A) Panoramic view of tumor sections. Scale bar: 3000 μm. (B) Magnified view of the tumor section. Scale bar: 50 μm.

**Figure S27**. Normalized ion image of **RhM** at 12 h post tail-vein injection of **RhM-R837@Lip** in 4T1 tumor sections measured by Desorption Electrospray Ionization–Mass Spectrometry Imaging (DESI-MSI). (A) **RhM** ion image at *m/z* 595.2937. (B) Mass spectrum of pixels shown in Figure (A).

**Figure S28**. Photographs of 4T1 tumor-bearing mice subjected to different treatments during phototherapy experiment from day 8 to day 18. (+) indicates laser irradiation (808 nm, 0.5 W/cm²).

**Figure S29**. Representative H&E staining images of tumors collected from mice after different treatments. (+) indicates laser irradiation (808 nm, 0.5 W/cm²). Scale bars: 200 µm.

**Figure S30**. Representative Ki-67 staining images of tumors collected from mice after different treatments. (+) indicates laser irradiation (808 nm, 0.5 W/cm²). Scale bars: 100 µm.

**Figure S31**. Analysis of CRT and HMGB1 expression in tumor sections collected from different treatment groups. Blue: DAPI-stained nuclei. Red: Immunofluorescence staining of CRT or HMGB1. Blue channel: Ex = 359 nm, Em = 457 nm. Red channel: Ex = 557 nm, Em = 570 nm. Scale bar: 100 μm.

**Figure S32**. Analysis of infiltrating CD3^+^/CD4^+^T cells and CD3^+^/CD8^+^T cells in 4T1 tumor sections collected from different treatment groups. Blue: DAPI-stained nuclei. Green: CD3^+^ antibody staining. Red: anti-CD4^+^ or anti-CD8^+^antibody staining. Blue channel: Ex = 359 nm, Em = 457 nm. Green channel: Ex = 491 nm, Em = 516 nm. Red channel: Ex = 557 nm, Em = 570 nm. Scale bar: 100 μm.

**Figure S33**. *In vivo* systemic anti-tumor immune response triggered by RhM-R837@Lip. (A) Flow cytometry results of CD4^+^ T cells in tumor from each group. (B) Flow cytometry analysis of CD8^+^ T cells in tumor from each group. (C) Percentage of CD4^+^ T cells in tumors. (D) Percentage of CD8^+^ T cells in tumors. Data are presented as mean ± SD (n = 3). Statistical significance was determined by Student’s *t*-test: *p < 0.05, **p < 0.01, ***p < 0.001.

**Figure S34**. Immunofluorescence analysis of infiltrating Treg cell in 4T1 tumor sections from different treatment groups. Blue: DAPI-stained nuclei. Green: anti-CD4 antibody staining (CD4⁺ cells). Yellow: anti-CD25 antibody staining (CD25⁺ cells). Red: anti-Foxp3 antibody staining (Foxp3⁺ cells). Blue channel: Ex = 359 nm, Em = 457 nm. Green channel: Ex = 491 nm, Em = 516 nm. Red channel: Ex = 557 nm, Em = 570 nm. Yellow channel: Ex = 656 nm, Em = 670 nm. Scale bar: 100 μm.

**Figure S35**. Quantification of inflammatory cytokines in 4T1 tumor tissues after different treatment: (A) TNF-α and (B) IFN-γ.

**Figure S36**. Immunohistochemical staining of PD-L1 in 4T1 tumor sections from different treatment groups. Scale bar: 100 μm.

**Figure S37**. Post-phototherapy serum biochemical analysis of liver and kidney function markers in mice from different treatment groups. (A) ALT levels. (B) AST levels. (C) ALP levels. (D) γ-GT levels. (E) BUN levels. (F) CR levels. (G) UA levels.

**Figure S38.** H&E-stained images of the heart, liver, spleen, lung, and kidney from 4T1 tumor-bearing BALB/c mice after treatment with different groups. Scale bars: 200 µm.

**Notes and References**

1. Liu, D.; Fu L.; Gong L.; Li S.; Li K.; Liu K.; Yang D., Proton-gradient-driven porphyrin-based liposome remote-loaded with imiquimod as in situ nanoadjuvants for synergistically augmented tumor photoimmunotherapy. *ACS Appl. Mater. Interfaces* **2024**, *16*, 8403−8416.
